# Endothelial KLF4 depletion drives age-related neurovascular dysfunction and neuropsychiatric impairment

**DOI:** 10.1073/pnas.2426990123

**Published:** 2026-06-18

**Authors:** Matasha Dhar, Edwin Vázquez-Rosa, Kalyani Chaubey, Emiko Miller, Sofia G. Corella, Suwarna Chakraborty, Sunil Jamuna Tripathi, Tapatee Das, Xudong Liao, Mohamed Alkassem Alosman, Hua Fang, Yeojung Koh, Preethy S. Sridharan, Kathryn Franke, Coral J. Cintrón-Pérez, Adrian A. Cintrón-Pérez, Taylor Tomco, Vidya Indrakumar, Phoebe J. Rubin, Justin G. Pieper, Luke A. Ashiku, Min-Kyoo Shin, Xinmiao Tang, Roshan Padmanabhan, Hariprakash Haragopal, Margaret E. Flanagan, Rajan Jain, Bradley D. Winters, Brigid M. Wilson, Bindu D. Paul, Mukesh K. Jain, Andrew A. Pieper

**Affiliations:** ^a^ Department of Psychiatry, Case Western Reserve University, Cleveland, OH 44106; ^b^ Brain Health Medicines Center, Harrington Discovery Institute, University Hospitals Cleveland Medical Center, Cleveland, OH 44106; ^c^ Geriatric Psychiatry, Geriatric Research Education and Clinical Center, Louis Stokes VA Medical Center, Cleveland, OH 44106; ^d^ Institute for Transformative Molecular Medicine, School of Medicine, Case Western Reserve University, Cleveland, OH 44106; ^e^ Department of Neurosciences, Case Western Reserve University, Cleveland, OH 44106; ^f^ Department of Physiology, Pharmacology and Therapeutics, Johns Hopkins University School of Medicine, Baltimore, MD 44106; ^g^Department of Molecular Biology, Cell Biology and Biochemistry, Division of Biology and Medicine, Brown University, Providence, RI 02912; ^h^Hathaway Brown School, Shaker Heights, OH 44122; ^i^ Massachusetts Institute of Technology, Cambridge, MA 02139; ^j^ Department of Pathology, Case Western Reserve University, Cleveland, OH 44106; ^k^ University of California, Santa Barbara, CA 93106; ^l^Northeastern University, Boston, MA 02115; ^m^Cate School, Carpinteria, CA 93013; ^n^ College of Pharmacy and Research Institute of Pharmaceutical Sciences, Seoul National University, Seoul 08226, Republic of Korea; ^o^ Yueyang Hospital, Shanghai University of Traditional Chinese Medicine, Shanghai, China; ^p^Trailhead Biosystems Inc., Beechwood, OH 44122; ^q^ Department of Biomedical Sciences, Northeast Ohio Medical University, Rootstown, OH 44272; ^r^ Glenn Bigg’s Institute for Alzheimer’s and Neurodegenerative Diseases, University of Texas Health Science Center at San Antonio, San Antonio, TX 78249; ^s^Department of Pathology, UT Health Science Center at San Antonio, San Antonio, TX 78249; ^t^Mesulam Center for Cognitive Neurology and Alzheimer’s Disease, Feinberg School of Medicine, Northwestern University, Chicago, IL 60611; ^u^Departments of Medicine and Cell and Developmental Biology, Penn Cardiovascular Institute, Penn Epigenetics Institute, Perelman School of Medicine, University of Pennsylvania, Philadelphia, PA 19104; ^v^ Division of Infectious Diseases and HIV Medicine in the Department of Medicine, Case Western Reserve University, Cleveland, OH 44106; ^w^ Department of Psychiatry and Behavioral Sciences, Johns Hopkins University School of Medicine, Baltimore, MD 21218; ^x^ The Solomon H. Snyder Department of Neuroscience, Johns Hopkins University School of Medicine, Baltimore, MD 21218; ^y^ Lieber Institute for Brain Development, Baltimore, MD 21218; ^z^Department of Molecular Biology, Cell Biology and Biochemistry, The Warren Alpert Medical School, Brown University, Providence, RI 02912

**Keywords:** blood–brain barrier, aging, neurodegeneration, KLF4, endothelial cell

## Abstract

Deterioration of the blood–brain barrier (BBB), including impaired neurovascular uncoupling, contributes to cognitive decline in aging. The BBB is formed principally by brain microvascular endothelial cells (ECs), and ECs throughout the body are enriched for the transcription factor Krüppel-like factor 4 (KLF4). Because KLF4 levels in ECs decrease with age, we tested whether that decline contributes to aging-related BBB deterioration, neurovascular dysfunction, and cognitive impairment. Using EC-specific *Klf4* knockout mice (EC-K4KO), we show that loss of EC KLF4 accelerates multiple age-related brain pathologies. Indeed, middle-aged EC-K4KO mice display pathological features that are not normally observed until advanced age, including marked BBB leakage, impaired neurovascular coupling, loss of microvessels, increased oxidative damage, neuroinflammation, neurodegeneration, anxiety-like behavior, and cognitive deficits. Single-cell RNA sequencing of brain vasculature reveals dysregulation of immune response and barrier-related genes in ECs lacking KLF4, indicating that KLF4 maintains brain endothelial homeostasis by constraining proinflammatory and senescence programs at the chromatin level. Together, these results identify loss of EC KLF4 as a key driver of neurovascular decline and age-associated cognitive dysfunction.

## Significance

We show that specific loss of KLF4 from endothelial cells advances the onset and severity of blood–brain barrier (BBB) breakdown, a key aspect of brain aging. The ensuing vascular and neuronal pathology leads to cognitive decline. Thus, mice with endothelial cell–specific elimination of KLF4 provide a readily applicable model of brain microvascular dysfunction in aging. Targeting endothelial KLF4 or its downstream pathways could offer a strategy to protect the brain from age-related BBB deterioration and cognitive impairment.

The blood–brain barrier (BBB) preserves neural homeostasis by regulating molecular exchange with the circulation and coupling cerebral blood flow to neuronal activity (neurovascular coupling) (1, 2). Early impairment in BBB function is a feature of age-related cognitive decline (3–7) and contributes to neurodegeneration in Alzheimer’s disease (AD) and traumatic brain injury (TBI) (8). Brain microvascular endothelial cells (ECs) form the vascular component of the neurovascular unit (9), are long-lived, and are highly responsive to cytokines, hormones, and trophic factors (4, 10–13), making them vulnerable to cumulative, age-related stress (13). Aging also remodels EC transcriptional programs, increasing inflammatory and oxidative pathways while downregulating BBB-maintaining genes (14), which impairs the regulation of vascular tone and flow, raises permeability, promotes hypoxia, and permits inappropriate plasma protein entry that harms brain health. However, the critical molecular regulators driving age-associated brain EC dysfunction remain incompletely defined.

Krüppel-like factor 4 (KLF4) is a zinc-finger transcription factor implicated in vascular biology and aging (15) that supports an anti-inflammatory, antithrombotic endothelial phenotype (16–21). KLF4 is abundant in brain ECs in mice (*SI Appendix*, Fig. S1*A*) (22) and humans (*SI Appendix*, Fig. S1*B*) (23) but declines with age (16), suggesting that EC KLF4 loss may drive BBB breakdown and neurovascular dysfunction. To test this, we characterized inducible, EC-specific KLF4 knockout (EC-K4KO) mice using cerebrovascular physiology, behavior, histology, and single cell transcriptomics. EC-K4KO mice develop accelerated, age-like cerebrovascular pathology, including BBB leakage, microvascular loss, and impaired neurovascular coupling, with increased oxidative damage, neuroinflammation, neurodegeneration, anxiety-like behavior, and cognitive deficits. Single-cell RNA sequencing of ECs from EC-K4KO brains revealed dysregulated immune response and BBB-related gene programs, underscoring KLF4’s central role in endothelial and neurovascular integrity. These results indicate that preserving EC KLF4 activity may be a viable strategy to prevent age-associated BBB deterioration and cognitive decline.

## Results

### EC KLF4 Loss Accelerates Age-Related Neurobehavioral and Cognitive Decline.

We generated EC-specific *Klf4* knockout (EC-K4KO) mice by crossing CDH5-CreERT2 with *Klf4*-flox mice, inducing Cre-induction at 8 wk of age as previously validated (17, 18, 24–26) (*SI Appendix*, Fig. S1*C*). Cohorts were assessed at young (4 to 6 mo), middle-aged (10 to 12 mo), and old (20 to 23 mo) time points. Sex-specific results are reported when observed; otherwise data were pooled.

**Fig. 1.**
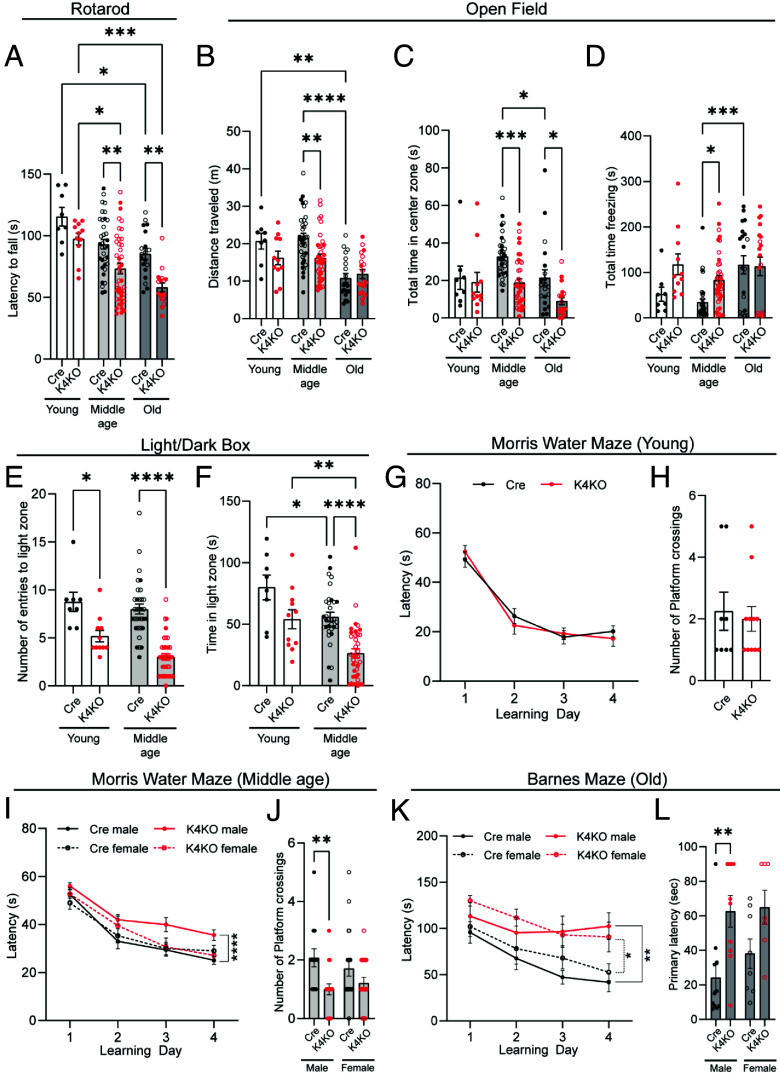
EC KLF4 depletion accelerates age-associated neurobehavioral and cognitive impairment. (*A*) Latency to fall on the test day of the accelerating rotarod test decreases with age in control mice (young WT Cre vs old WT Cre), while this decrease is accelerated in EC-K4KO mice (young EC-K4KO vs middle-aged EC-K4KO and Young EC-K4KO vs old EC-K4KO) and worsened in EC-K4KO mice when compared to middle-aged and old-aged WT Cre littermates. (*B*) Distance traveled in the open field is reduced with age in control mice (young WT Cre vs old WT Cre and middle-aged WT Cre vs old WT Cre) while EC-K4KO mice show deficits only in middle-age (compared to middle-aged WT Cre). (*C*) Old WT Cre mice spend less time in the center zone of the open field than middle-aged WT Cre mice. EC-K4KO mice in both age groups spend less time in the center zone than WT Cre littermates. (*D*) Old WT Cre mice spend more time freezing in the open field than middle-aged WT Cre mice. EC-K4KO mice in both age groups spend more time freezing than WT Cre littermates. (*E*) Young and middle-aged EC-K4KO mice make fewer entries into the light zone of the light/dark box than WT Cre littermates. (*F*) Middle-aged WT Cre mice spend less time in the light zone of the light/dark box than young WT Cre mice. Both young and middle-aged EC-K4KO mice spend less time in the light zone of the light/dark box than WT Cre littermates. Learning (*G*) and memory (*H*) in the Morris water maze were not affected in young EC-K4KO mice. (*I*) Learning and memory (*J*) in the Morris water maze were impaired in middle age male EC-K4KO mice, compared to WT Cre littermates, but not in females. (*K*) Learning in the Barnes maze was impaired in both male and female EC-K4KO mice, compared to WT Cre littermates. (*L*) Memory in the Barnes maze was impaired in male EC-K4KO mice, compared to WT Cre littermates, and females showed a similar trend. Data are average (±SEM). Data points represent individual animals with black filled circles as WT Cre males, black open circles as WT Cre females, red closed circles as EC-K4KO males, and red open circles as EC-K4KO females (except panels *G*, *I*, and *K*, which are average values). Significance was tested using one-way ANOVA and Bonferroni post hoc analysis for all panels except panels *G*, *I*, and *K*, which were analyzed using two-way ANOVA and Bonferroni post hoc analysis. n = 8 Young Cre, n = 11 Young K4KO, n = 14 middle-aged Cre males, n = 20 middle-aged Cre females, n = 20 middle-aged K4KO male, n = 20 middle-aged K4KO female, n = 11 Old Cre male, n = 8 Old Cre female, n = 10 Old K4KO male, n = 7 Old K4KO female. *P < 0.05, **P < 0.01, ***P < 0.001, ****P < 0.0001

In the accelerating rotarod test, wild-type (WT) Cre controls showed the expected age-related decline in latency to fall (young vs old WT Cre) (Fig. 1*A*). EC-K4KO mice exhibited accelerated impairment, with significantly reduced performance by middle-age versus WT Cre littermates (Fig. 1*A*). Grip strength was unchanged (*SI Appendix*, Fig. S2*A*), indicating a neurologic rather than muscular deficit. In the open field, total distance declined with age in WT Cre mice, and middle-aged EC-K4KO mice showed further reductions relative to age-matched WT Cre littermates (Fig. 1*B*).

Anxiety-like behavior increased with age in WT Cre mice, evidenced by reduced center time (Fig. 1*C*), increased freezing (Fig. 1*D*), fewer center entries (*SI Appendix*, Fig. S2*B*), and greater border time (*SI Appendix*, Fig. S2*C*). EC-K4KO mice displayed greater anxiety-like behavior on these measures at middle and old ages versus WT Cre littermates (Fig. 1 *C* and *D*). In the light/dark box, young and middle-aged EC-K4KO mice made fewer entries and spent less time in the light zone than WT Cre littermates (Fig. 1 *E* and *F*). Middle-aged WT Cre mice also spent less time in the light zone than young WT Cre mice (Fig. 1 *E* and *F*).

Cognitive testing showed no impairment in young EC-K4KO mice in the Morris water maze (27) (Fig. 1 *G* and *H* and *SI Appendix*, Fig. S2*D*). Middle-aged male EC-K4KO mice exhibited significant spatial learning and memory deficits versus WT Cre littermates, while females trended similarly but did not reach significance (Fig. 1 *I* and *J*). Swim speeds were comparable across groups (*SI Appendix*, Fig. S2 *E* and *G*). Although platform crossings differed at middle age (Fig. 1*J*), time in the target zone did not (*SI Appendix*, Fig. S2*F*). For older mice, we used the Barnes maze (28) to eliminate challenges of swimming. Male and female EC-K4KO mice showed impaired learning and memory versus WT Cre littermates (Fig. 1 *K* and *L* and *SI Appendix*, Fig. S2*H*), with no travel speed differences (*SI Appendix*, Fig. S2*I*).

In summary, EC KLF4 deletion accelerates age-associated deficits in motor coordination, anxiety regulation, and hippocampal-dependent cognition.

### EC KLF4 Loss Accelerates BBB Deterioration and Neurovascular Dysfunction.

**Fig. 2.**
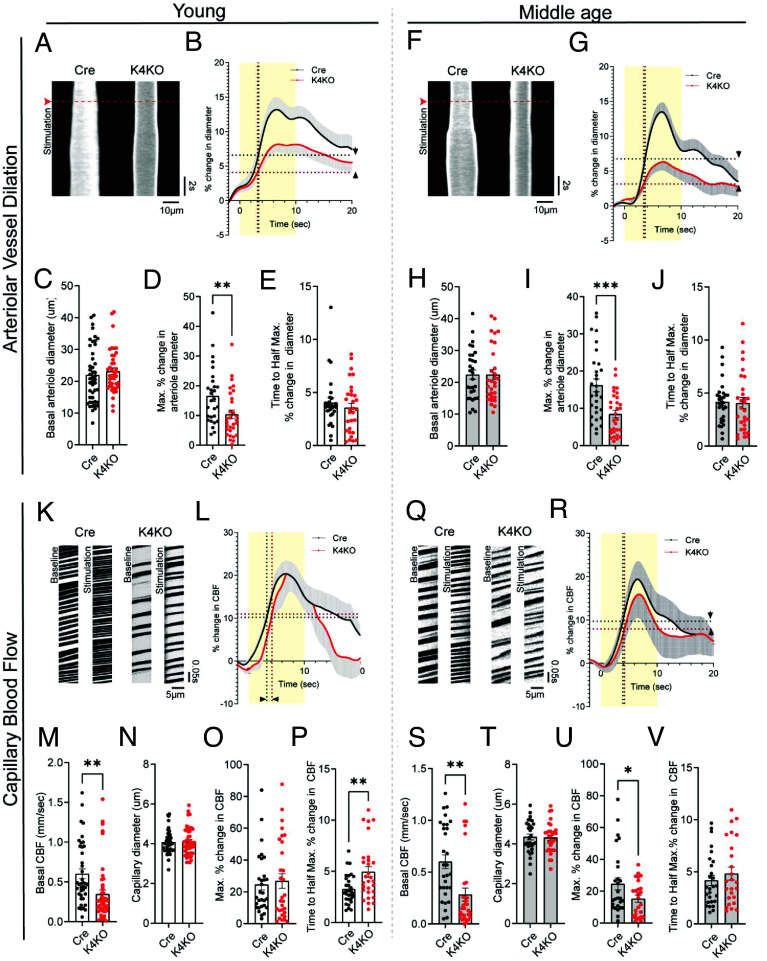
EC KLF4 depletion accelerates neurovascular dysfunction. (*A* and *B*) Representative two-photon line-scan images and average time course of arteriolar dilation before and after hind limb stimulation in young EC-K4KO and WT Cre littermates (n = 30 arterioles/5 mice for young EC-K4KO mice and 28 arterioles/4mice for young WT Cre littermates). The red dashed line in (*A*) indicates the start of stimulation. The yellow highlighted section in (*B*) indicates the entire 10 s stimulation duration. Horizontal red (EC-K4KO) and black (WT Cre littermates) dashed lines indicate half-max vasodilation, and vertical red (E-K4KO) and black (WT Cre littermates) dashed lines indicate time to half-max vasodilation. Arrowheads indicate decrease in half-max vasodilation. (*C*) Basal arteriolar diameter was not different between groups. (*D*) Maximum % change in arteriolar diameter in response to stimulation was significantly reduced in young EC-K4KO mice. (*E*) Time to half maximum % change in diameter was not different between groups. (*F* and *J*) Representative two-photon line-scan images (*F*) and average time course of arteriolar dilation (*G*) before and after hind limb stimulation in middle-aged WT Cre and middle-aged EC K4KO (n = 30 arterioles for each of 4 mice for middle-aged EC-K4KO and WT Cre littermates). Maximum % change in arteriolar diameter in response to stimulation was reduced in middle-aged EC-K4KO mice (*I*) without any difference in basal arteriolar diameter (*H*) or time to half maximum % change in diameter (*J*), compared to WT Cre littermates. Representative two-photon line-scan images (*K*) and average time course of capillary blood flow (CBF) (*L*) during baseline and after hind limb stimulation in young EC-K4KO and WT Cre littermates (n = 30 capillaries from each of 4 mice). In (*L*), the yellow highlighted section indicates the entire 10 s stimulation duration, horizontal black (WT Cre) and red (EC-K4KO) dashed lines indicate half-max increase in CBF, vertical black (WT Cre) and red (EC-K4KO) dashed lines indicate time to half-max CBF, and arrowheads indicate decrease in half-max CBF. Basal CBF (*M*) is reduced in young EC-K4KO mice, while capillary diameter (*N*) is the same between groups. Maximum % change in CBF (*O*) was the same between groups while the time to half maximum % change in CBF (*P*) was slower in young EC-K4KO mice. Representative two-photon line-scan images (*Q*) and average time course of capillary blood flow (*R*) during baseline and after hind limb stimulation in middle-aged EC-K4KO and WT Cre littermates (n = 25 capillaries from each of 4 EC-K4KO mice and n = 27 capillaries from each of 4 WT Cre littermates). Basal CBF (*S*) remained slower in middle-aged EC-K4KO mice without any difference in capillary diameter (*T*) or time to half maximum % change in CBF (*V*). (*U*) Middle-aged EC-K4KO mice showed a reduced maximum % change in CBF. Data are average (±SEM). Data points represent individual blood vessels with red closed circles for EC-K4KO mice and black filled circles for their WT Cre littermates. Significance was tested using two-tailed *t* test. *P < 0.05, **P < 0.01, ***P < 0.001, ****P < 0.0001

Because ECs maintain the BBB, we assessed whether cognitive deficits in EC-K4KO mice were associated with accelerated BBB and neurovascular decline using in vivo two-photon microscopy to measure arteriolar and capillary function, tracer leakage, and ultrastructure. Basal arteriolar diameter was unchanged in young EC-K4KO mice versus WT Cre littermates (Fig. 2 *A* and *C*). Hind limb stimulation produced robust arteriolar dilation in WT Cre mice that was markedly attenuated in young EC-K4KO mice (Fig. 2 *B* and *D*), indicating impaired neurovascular coupling. Middle-aged EC-K4KO mice similarly showed reduced stimulus-evoked arteriolar dilation with unchanged basal diameter (Fig. 2 *F*–*I*). Time to half-maximal dilation was comparable across groups (Fig. 2 *E* and *J*), consistent with reduced maximal dilatory capacity after KLF4 loss.

Because microvascular responses drive upstream vasodilation (1), we examined neurovascular responses in capillaries (< 6 µm in diameter). Young and middle-aged EC-K4KO mice had lower baseline capillary blood flow (CBF) than WT Cre littermates (Fig. 2 *K*, *M*, *Q*, and *S*). Young EC-K4KO mice achieved a similar percent increase in activity-induced CBF as WT Cre littermates but with delayed kinetics (Fig. 2 *K*–*P*). Middle-aged EC-K4KO mice showed further impairment with a reduced activity-induced percent increase, while time to half-maximal CBF was unchanged (Fig. 2 *Q–V*).

**Fig. 3.**
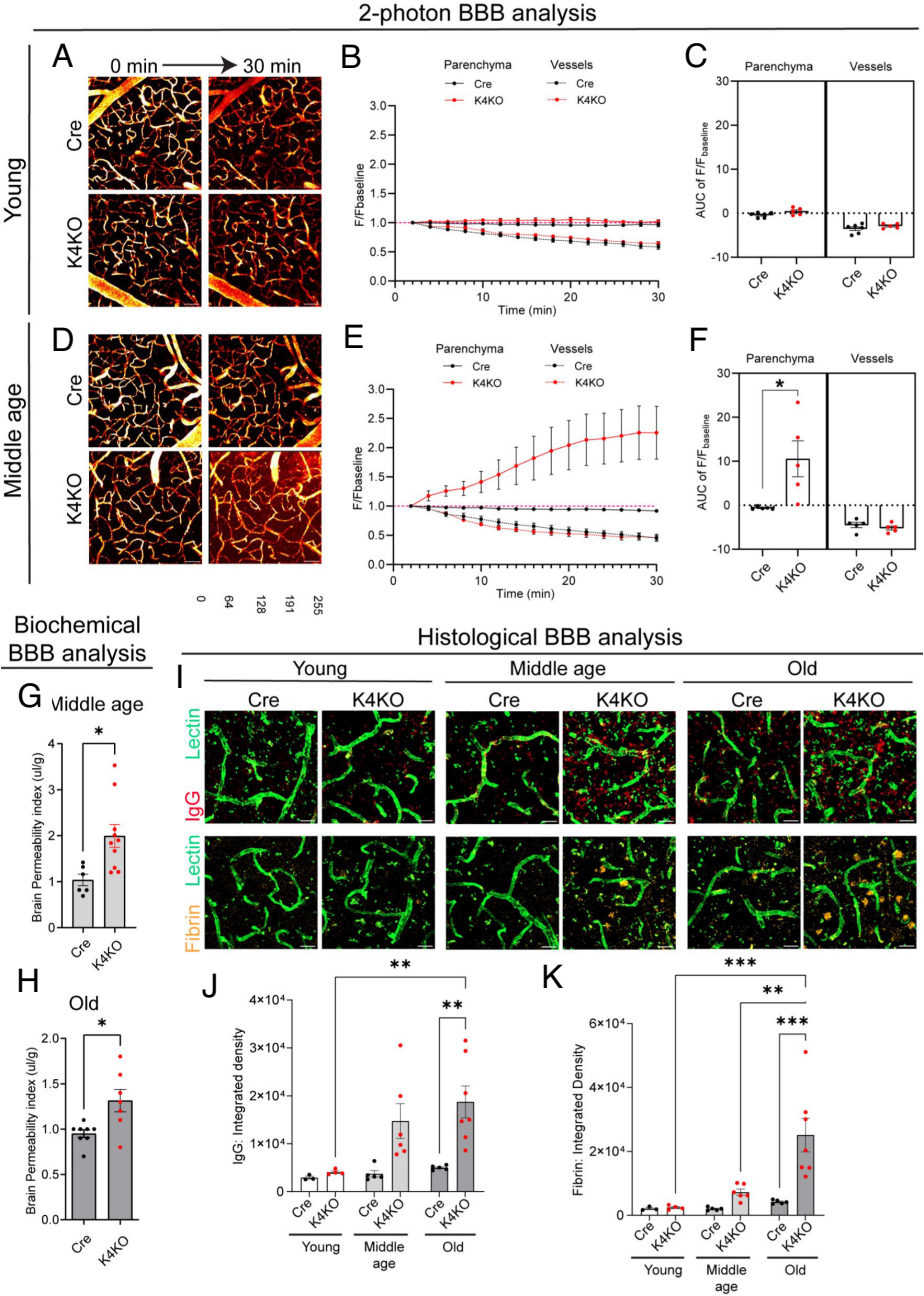
EC KLF4 depletion disrupts the BBB after middle-age. (*A* and *D*) Representative two-photon z-projection images from the same field of view 2 and 30 min after retro-orbital injection of 3kD fluorescent-dextran in young (*A*) and middle-aged (*D*) EC-K4KO and WT Cre littermate mice. Average time course of change in parenchymal and vessel fluorescent intensity shows no increase in parenchymal fluorescence in young EC-K4KO or WT Cre littermate mice (*B*). There is a robust increase in parenchymal fluorescent intensity in middle-aged EC-K4KO mice compared to WT Cre littermates (*D* and *E*). All groups showed an equivalent decrease in signal intensity over time. (*C* and *F*) Area under the curve (AUC) was calculated from the time course data showing significant parenchymal accumulation of 3kD fluorescent-dextran in middle-aged EC-K4KO mice. BBB permeability to 3kD fluorescent-dextran was measured using a biochemical assay in middle-aged (*G*) and old (*H*) mice. (*I*) BBB permeability to endogenous plasma proteins, IgG (red, *Top* row), and fibrin (orange, *Bottom* row), was measured using immunohistochemical assay. Lectin (green) was used to stain blood vessels. Representative images and quantified data are from the mouse cortex. Cortical parenchymal IgG (*J*) and fibrin (*K*) levels are significantly increased with age in EC-K4KO mice and significantly increased in old EC-K4KO mice compared to WT Cre littermates. Data shown are average (±SEM). Data points represent individual animals with black filled circles as WT Cre and red closed circles as EC-K4KO. Significance was tested using two-tailed *t* test for panels *C*, *F*, *G*, and *H* and one-way ANOVA and Bonferroni post hoc analysis for panels *J* and *K*. *P < 0.05, **P < 0.01, ***P < 0.001, ****P < 0.0001

We assessed BBB permeability with retro-orbitally injected 3kD fluorescent dextran and two-photon microscopy. This assay indicates advanced structural BBB disruption capable of permitting entry of plasma proteins, large molecules, and immune cells into the brain parenchyma. This contrasts with lower molecular weight tracers, which more sensitively detect early or mild BBB disruption, for example in response to subtle tight junction loosening or increased EC transcytosis. No parenchymal dextran accumulation occurred in young mice of either genotype (Fig. 3 *A*–*C*). By middle age, EC-K4KO mice showed significantly greater parenchymal dextran accumulation than WT Cre littermates, indicating increased BBB leakage (Fig. 3 *D*–*F*). Whole brain biochemical assays confirmed an elevated BBB permeability index in EC-K4KO mice at middle and old ages (Fig. 3 *G* and *H*). Histology of old mice revealed increased parenchymal fibrin and IgG in EC-K4KO brains (Fig. 3 *I*–*K*).

Transmission electron microscopy across ages showed progressive BBB structural deterioration in EC-K4KO mice versus WT Cre littermates. Astrocytic endfeet swelling was more frequent in EC-K4KO mice relative to WT Cre littermates at young, middle, and old ages, and capillary breaks that were observed in old EC-K4KO mice were absent in WT Cre littermates (*SI Appendix*, Fig. S3 *A* and *B*). Middle-aged EC-K4KO mice also had reduced claudin 5 expression, with no change in ZO-1 or occludin (*SI Appendix*, Fig. S3 *C* and *D*).

Thus, EC KLF4 depletion impairs neurovascular coupling, reduces capillary perfusion, increases BBB permeability, and accelerates progressive BBB structural damage, demonstrating that EC KLF4 is required to preserve neurovascular and barrier integrity with age.

### EC KLF4 Loss Accelerates Neuroinflammation and Oxidative Brain Damage.

**Fig. 4.**
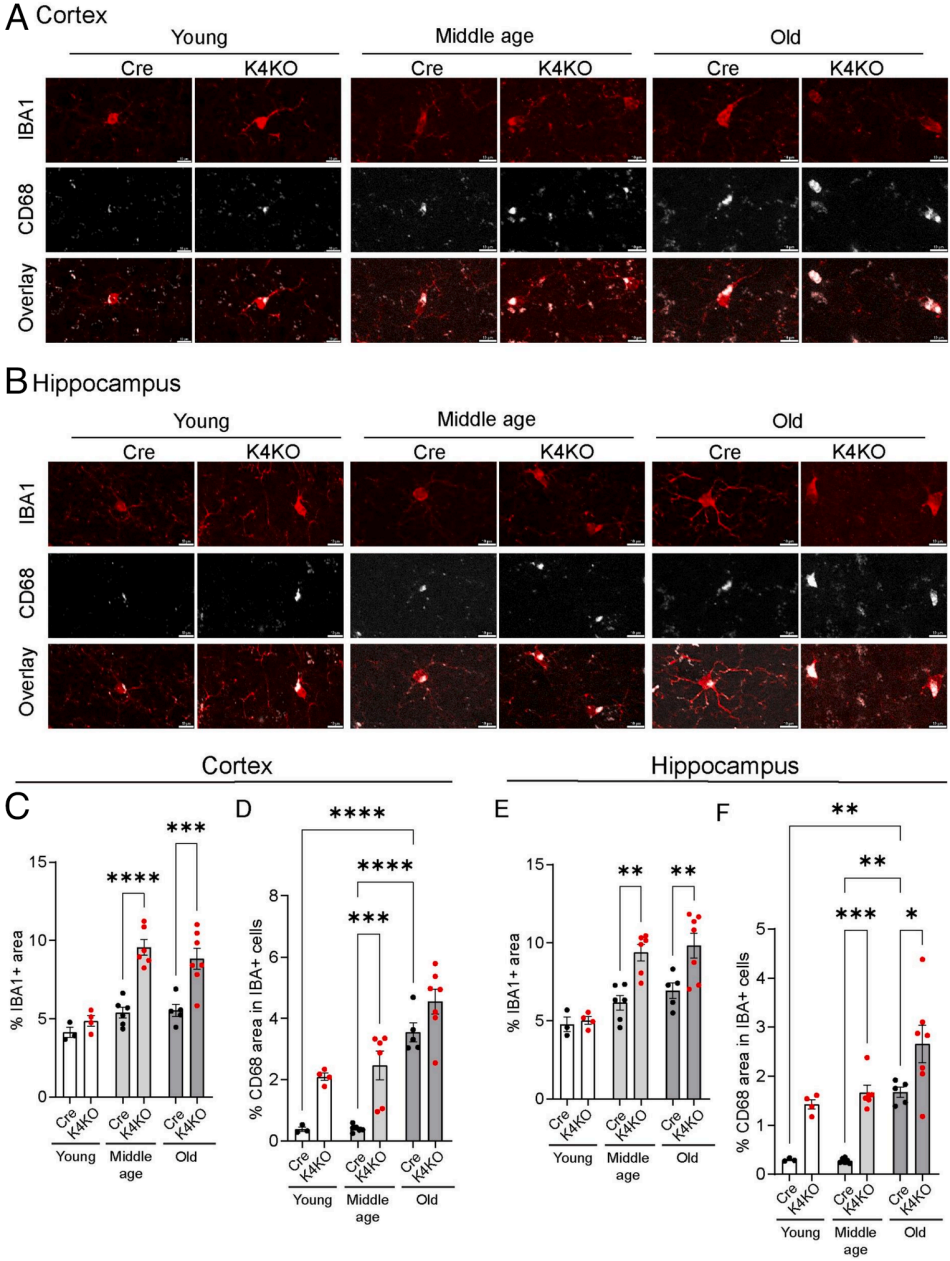
EC KLF4 depletion accelerates neuroinflammation, (*A* and *B*) Immune activation was measured using immunohistochemical staining for IBA1 (red) and CD68 (white) in the cortex (*A*) and hippocampus (*B*). (*C*–*F*) Quantification of % IBA1 area (*C* and *E*) and % CD68 area in IBA1+ cells (*D* and *F*) show increased immune activation in the cortex and hippocampus of middle-aged EC-K4KO mice. Old WT Cre mice also show increased %CD68 in IBA1+ cells (*D* and *F*), compared to young WT Cre. Data are average (±SEM). Data points represent individual animals with black filled circles as WT Cre and red closed circles as EC-K4KO. Significance was tested using one-Way ANOVA and Bonferroni post hoc analysis. *P < 0.05, **P < 0.01, ***P < 0.001, ****P < 0.0001

Given age-associated increases in neuroinflammation (29–31), we tested whether this was accelerated in EC-K4KO mice. Ionized calcium-binding adaptor molecule 1 (IBA1) labeling, a marker of microglial morphology and activation (32), showed increased IBA1-positive area in the cortex and hippocampus of middle-aged and old EC-K4KO mice versus WT Cre littermates (Fig. 4 *A*–*F*). Costaining for CD68, a microglial phagocytic marker that rises with age (14, 33, 34), revealed a greater proportion of IBA1+ cells that were CD68+ in middle-aged and old EC-K4KO mice (Fig. 4 *A*–*F*), indicating acceleration of aging-related microglial activation. Old WT Cre mice showed increased Cd68+:Iba1+ microglia versus young WT Cre mice (Fig. 4 *A*, *B*, and *F*).

Neuroinflammation combined with BBB compromise promotes oxidative damage (35), and aging correlates with reduced antioxidant defenses (36–43). The brain’s high oxygen consumption and lipid-rich neuronal membranes make it especially vulnerable to lipid peroxidation (44), resulting in increased levels of aldehyde 4-hydroxy-2-nonenal (4-HNE), a marker of lipid peroxidation that accumulates with brain aging (45–47). We observed elevated cortical 4-HNE in old WT Cre mice versus middle-aged WT Cre mice, and progressively higher 4-HNE in EC-K4KO mice compared with WT Cre littermates at middle and old ages (*SI Appendix*, Fig. S4 *A* and *E*). Similar increases occurred in CA3 and dentate gyrus (DG) of middle-aged EC-K4KO mice (Fig. 3 *B* and *F*).

Nitrosative stress, assessed by measuring levels of the 3-nitrotyrosine (3-NT) aging-associated protein modification (48–55), rose in old WT Cre mice versus middle-aged WT Cre mice and increased progressively in EC-K4KO mice relative to WT Cre littermates across all ages (*SI Appendix*, Fig. S4 *C* and *G*). Middle-aged EC-K4KO mice showed elevated 3-NT in CA3 and DG relative to WT Cre littermates (Fig. 3 *D* and *H*).

Collectively, our results show that EC KLF4 loss accelerates aging-related BBB deterioration, microglial activation, and oxidative/nitrosative damage.

### EC KLF4 Loss Accelerates Microvascular Loss and Axonal Degeneration.

**Fig. 5.**
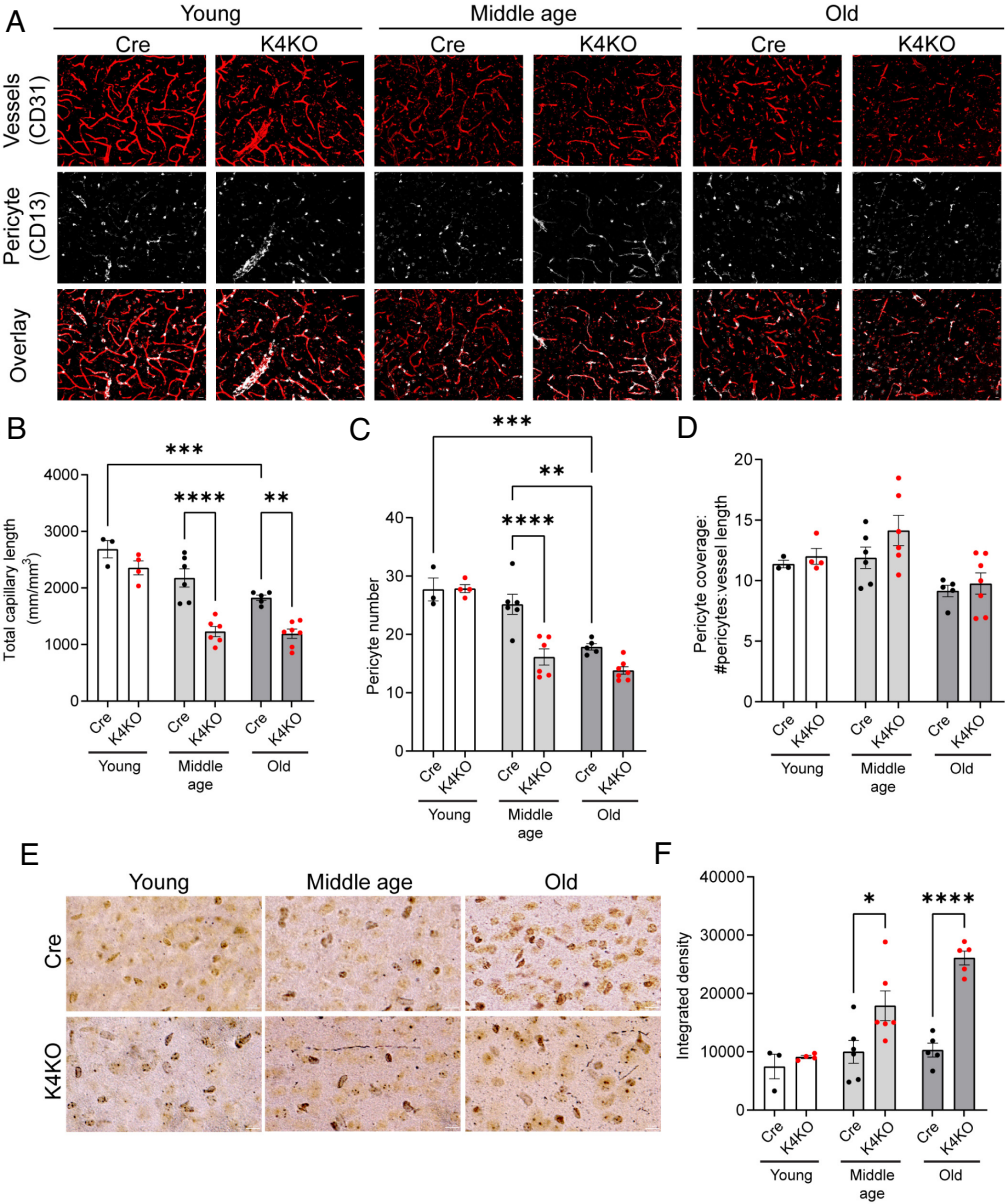
EC KLF4 depletion causes vessel loss and axonal degeneration in the cortex beginning at middle-age. (*A*) Vessel health was measured using immunohistochemical staining for endothelial cell marker (CD31, red) and pericyte marker (CD13, white). Representative images and quantified data are from the mouse cortex. (*B*) Reduction in total capillary length is seen with age in WT Cre littermates (old WT Cre vs young WT Cre), while loss of EC KLF4 significantly reduces vessel length by middle-age (middle-aged EC-K4KO vs middle-aged WT Cre littermate), which is sustained through old-age (old EC-K4KO vs old WT Cre littermate). (*C*) Reduction in pericyte number is seen analogous to reduction in capillary length. (*D*) Pericyte coverage of capillaries does not change in any group. (*E* and *F*) Axonal degeneration was measured by silver staining in each group. Representative images and quantified data are from the mouse cortex. A significant increase in axonal degeneration, as quantified by integrated density of silver deposits, is seen in middle-aged and old K4KO mice, relative to Cre littermates. Data are average (±SEM). Data points represent individual animals with black filled circles as Cre and red closed circles as K4KO. Significance was tested using One-Way ANOVA and Bonferroni post hoc analysis. *P < 0.05, **P < 0.01, ***P < 0.001, ****P < 0.0001

Because the BBB supports vascular and neuronal integrity, we examined microvascular and axonal changes following endothelial KLF4 depletion. Immunostaining for CD31 (endothelium) and CD13 (pericytes) showed age-related reductions in total capillary length and pericyte number in WT Cre mice in the cortex (Fig. 5 *A*–*D*) and hippocampus (*SI Appendix*, Fig. S5 *A–D*). The hippocampus was affected earlier, with middle-aged WT Cre mice showing reductions versus young controls (*SI Appendix*, Fig. S5 *B* and *C*), consistent with reported hippocampal vulnerability (56–59).

In the WT Cre cortex, pericyte coverage of CD31+ vessels did not change with age but decreased in the hippocampus of old WT Cre mice (*SI Appendix*, Fig. S5*D*). EC-K4KO mice showed accelerated microvessel degeneration with decreased capillary length in the cortex (Fig. 5 *A* and *B*) and hippocampus (*SI Appendix*, Fig. S5 *A* and *B*) beginning at middle age and progressing into old age. Total pericyte number and pericyte coverage were not further reduced in EC-K4KO mice (Fig. 5 *C* and *D* and *SI Appendix*, Fig. S5 *C* and *D*).

Silver staining revealed increased axonal degeneration in middle-aged and old EC-K4KO mice versus WT Cre littermates, quantified by higher integrated density of silver deposits (Fig. 5 *E* and *F* and *SI Appendix*, Fig. S5 *E* and *F*). Thus, EC KLF4 deficiency accelerates age-related microvascular loss and axonal degeneration, pathologies that impair cognition independently of neuronal cell death (60–64).

### EC KLF4 Loss Alters the Brain Transcriptomic Landscape.

**Fig. 6.**
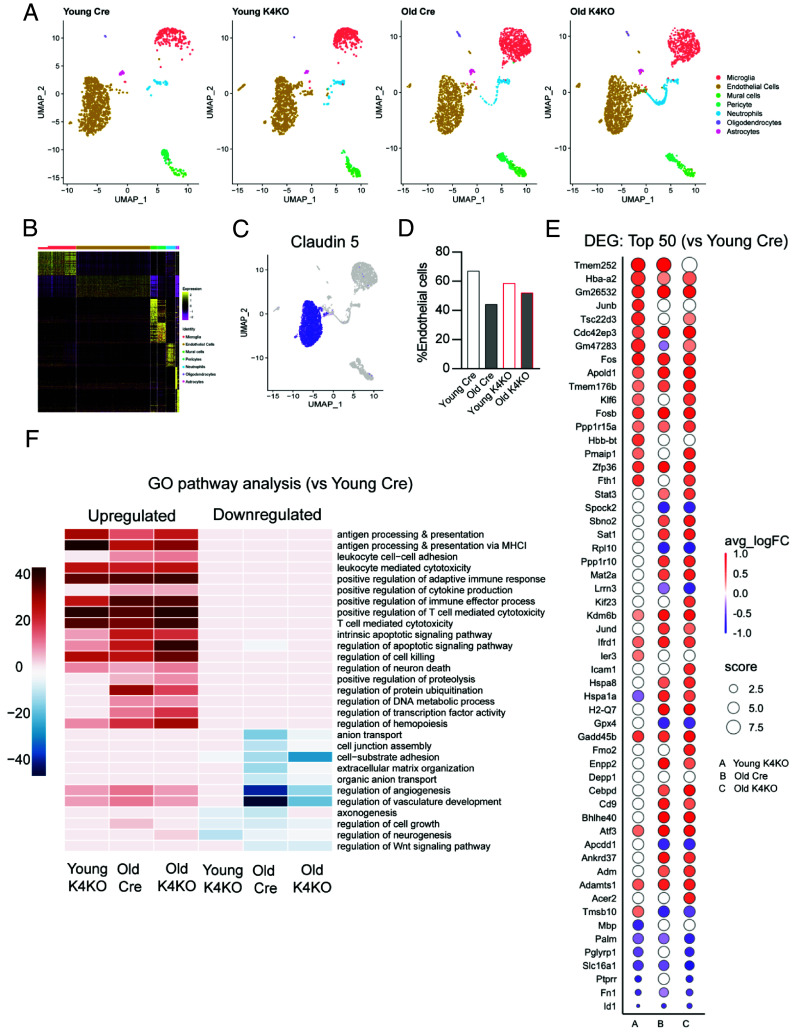
EC KLF4 depletion causes young EC-K4KO brain cells to develop an old-age transcriptional profile. (*A*) Uniform manifold approximation and projection for dimension reduction (UMAP) and unsupervised clustering analysis using Seurat pipeline identified seven distinct cell populations (endothelial cells, microglia, mural cells, pericytes, neutrophils, oligodendrocytes, astrocytes) from the total of 7398 cells (young WT Cre = 1,285 cells, young EC-K4KO = 1,358 cells, old WT Cre = 2,506 cells, old EC-K4KO = 2,249 cells). (*B*) Heatmap of the top 50 most enriched genes for each cell type identified by unbiased whole transcriptome clustering. (*C*) FeaturePlot depicting claudin 5 gene expression on UMAP is specific for the cluster identified as endothelial cells. (*D*) Comparison of percent of endothelial cells isolated from each group. (*E*) Dotted heatmap of top 50 DEGs expressed in endothelial cells in each group (compared to young WT Cre) ranked by the avg_logFC values. Color indicates the average logFC of the group (A: young EC-K4KO, B: old WT Cre, C: old EC-K4KO) in comparison to young WT Cre, while the dot size represents degree of statistical significance. (*F*) Gene ontology (GO) analyses with DEGs in endothelial cells from each group comparison shown in (*E*) showing the top 30 upregulated and downregulated biological process (BP) GO terms.

To define molecular mechanisms, we performed single cell RNA sequencing on young and old brains. Unsupervised clustering identified major brain cell types, including microglia, neutrophils, ECs, pericytes, astrocytes, oligodendrocytes, and mural cells (Fig. 6*A*, *B*). Neutrophil abundance was moderately but consistently increased in EC-K4KO brains at both ages, consistent with increased peripheral immune cell surveillance and potential parenchymal infiltration secondary to endothelial dysfunction.

We focused on the claudin 5 positive EC cluster, enriched for BBB-forming ECs and the largest EC subpopulation (Fig. 6*C*). Comparing the top 50 differentially expressed genes (DEGs) in this cluster from young EC-K4KO, old WT Cre, and old EC-K4KO (each compared to young WT Cre) revealed overlapping transcriptional changes (Fig. 6 *D* and *E*), indicating that EC KLF4 loss recapitulates aspects of the aged EC transcriptome. Pathway analysis of these DEGs showed upregulation of immune response and cell death pathways and downregulation of vascular and barrier integrity pathways (Fig. 6*F*). Thus, EC KLF4 deficiency drives a shift in BBB EC transcription toward an inflamed, degenerative state that mirrors aging.

### EC KLF4 Loss Alters Chromatin Accessibility and Transcription.

**Fig. 7.**
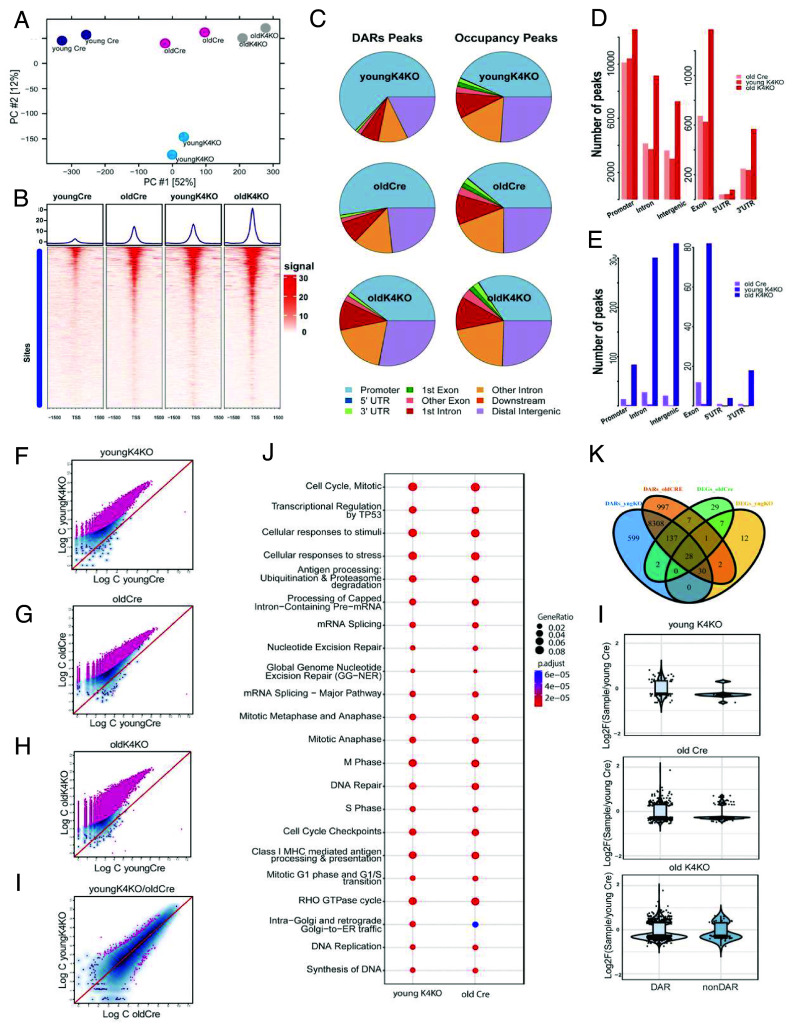
EC KLF4 depletion causes the young EC-K4KO brain to develop an old-age chromatin accessibility and transcription profile. (*A*) PCA plot of normalized sequence counts from ATAC-seq, with each colored circle representing biological replicates of four groups labeled as young WT Cre, old WT Cre, young EC-K4KO, and old EC-K4KO. (*B*) Heat maps of normalized sequence tags mapped with respect to transcription start site (TSS) (1.5 kb region) of all annotated protein coding gene conditions as mentioned in (*A*). (*C*) genomic features of regions that require chromatin accessibility (DA regions, ATAC-seq) (*D* and *E*). The distribution of differentially accessible regions (DARs) in different genomic coordinates: (*D*) depleted DARs and (*E*) enriched DARs. (*F*–*H*) Differential accessibility (DA) analysis of ATAC-seq from young WT Cre versus old WT Cre and young/old EC-K4KO. Blue dots indicate non-DA sites. Pink dots indicate significant (FDR < 0.001) differential peaks normalized to young WT Cre identified by DiffBind (DESeq2) (DA peaks). (*I*) DA analysis of peaks from old WT Cre and young EC-K4KO. The number of peaks and representative genes in each class is noted on the plot. (*J*) Dot plot of top 20 shared enriched GO terms of pathway for both DARs young K4KO and old Cre. (*K*) Venn diagram showing overlap peaks from DARs and DEG. (*L*) Violin plot comparing DEG to DA and non-DA of young WT Cre vs old WT Cre, with young and old EC-K4KO ranked by logFC values.

Single-cell transcriptomics indicated that ECs from young EC-K4KO mice adopt an aged-like program, suggesting an epigenetic component, and KLF4 has known roles in chromatin remodeling (65–67). We therefore performed genome-wide Omni-ATAC (assay for transposase-accessible chromatin using sequencing) (68) on purified brain EC nuclei. Principal component analysis (PCA) confirmed replicate consistency (Fig. 7*A*) and read-occupancy across protein-coding genes is summarized in Fig. 7*B*.

We identified DARs of chromatin encompassing all classes of cis-regulatory elements (including promoters, enhancers, and insulators) across old WT Cre, young EC-K4KO, and old EC-K4KO mice (all compared to young WT Cre). Most DARs in old WT Cre and young EC-K4KO mapped to promoters and distal intergenic regions (Fig. 7*C*). Regions with increased accessibility corresponded to upregulated genes and decreased accessibility matched downregulated genes relative to young WT Cre littermates (Fig. 7 *D* and *E*).

Both old WT Cre and young EC-K4KO mice showed similarly enriched DARs primarily in promoters and distal intergenic regions. DA analysis of ATAC seq comparing young WT Cre to old WT Cre, old EC-K4KO, and young EC-K4KO mice (Fig. 7 *F*–*H*) revealed fewer (indicated by blue dots) and more significant (FDR<0.001; indicated by pink dots) non-DA sites, normalized to young WT Cre and identified by DiffBind (DeSeq2). However, DA analysis between old WT Cre and young EC-K4KO mice revealed significant overlapping regions, indicating that EC KLF4 loss recapitulates chromatin changes seen with aging (Fig. 7*I*).

Pathway enrichment analysis for overlapping genes highlighted stress- and senescence-related pathways, including TP53 transcriptional regulation, cellular stress response, and cell cycle checkpoints (Fig. 7*J*). Integrating DARs with RNA-seq DEGs identified 173 genes overlapping with old WT Cre DARs and 61 genes overlapping with young EC-K4KO DARs (Fig. 7*K*). A violin plot comparing the different groups showed that most DEGs overlapped with DA regions (Fig. 7*L*), indicating the EC KLF4 loss recapitulates aging-associated chromatin changes.

**Fig. 8.**
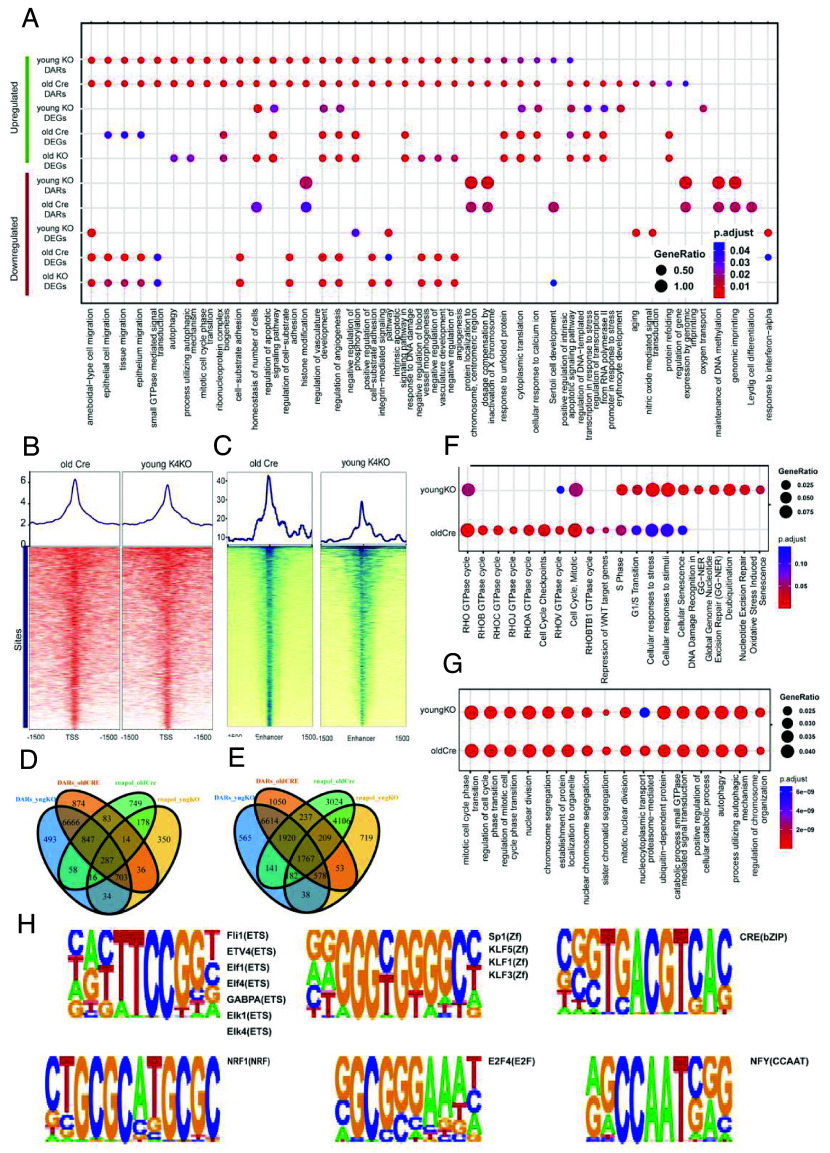
Integration of RNAseq with ATAC seq and ChIP seq in EC-K4KO brain cells. (*A*) Cluster dot plot of enriched GO terms for top 30 upregulated and downregulated biological processes for DEG and DAR of all the groups. (*B*) Heat maps of chromatin immunoprecipitation followed by sequencing reads of RNA PolII were plotted ±1,500 bp from the peak midpoint of TSS. (*C*) Venn diagram showing overlap peaks from RNA PolII ChIP Seq and DARs of ATAC seq from TSS. (*D*) Heat maps of chromatin immunoprecipitation followed by sequencing reads of RNA PolII were plotted ±1,500 bp from the peak midpoint of enhancers. (*E*) Venn diagram showing overlap peaks from RNA PolII ChIP Seq and DARs of ATAC seq from enhancer regions. (*F* and *G*) Cluster dot plot analysis by GO biological processes for common targets identified by integration of ChIP seq peaks to DAR peaks of young EC-K4KO and old WT Cre from TSS and enhancers, respectively. (*H*) common motif enrichment from RNA PolII ChIP Seq and DARs of ATAC seq.

Thus, EC KLF4 deficiency in young mice induces chromatin remodeling and transcriptional reprogramming that mirror aging, enriching stress- and senescence-associated pathways and linking DARs to corresponding DEGs (Fig. 8*A*). Importantly, these corresponded to relative levels of key proteins quantified by immunohistochemical staining in ECs (GADD34, ADAMTS1, and MCT1) (*SI Appendix*, Fig. *S6 A–E*).

### EC KLF4 Loss Alters Transcriptional Activity At Actively Transcribed Genes.

To validate RNA-seq and ATAC-seq, we performed chromatin immunoprecipitation sequencing for RNA polymerase II (Pol II) to profile active transcription. We compared young EC-K4KO and old WT Cre groups. Genome-wide Pol II occupancy at TSS (Fig. 8*B*) and enhancer-distal regions (Fig. 8*C*) showed similar overall patterns between groups.

Integrating Pol II ChIP-seq with ATAC-seq DARs mapped transcriptional activity to accessible chromatin for ~70% of genes (Fig. 8 *D* and *E*). GO enrichment of the overlapping gene set recapitulated the stress-, senescence-, and vascular integrity pathways identified in the DAR-DEG overlap (Fig. 8 *F* and *G*), reinforcing that EC KLF4 loss in young mice drives an aging-like transcriptional program.

Motif enrichment analysis of ATAC-seq and ChIP-seq revealed significant enrichment for ETS and zinc-finger family motifs in old WT Cre and young EC-K4KO samples (Fig. 8*H*), consistent with roles for these factors in endothelial regulation and cellular senescence (69).

## Discussion

Here, we identify EC KLF4 as a central regulator of microvascular integrity and brain health during aging. EC-specific KLF4 deletion was induced with tamoxifen (2 mg/25 g) and known side effects (local peritoneal irritation, weight loss, hepatotoxicity, reduced exploratory drive, bone marrow suppression) were controlled by treating control and experimental groups identically. We observed that EC KLF4 loss accelerates age-associated neurovascular dysfunction and BBB breakdown, producing neuroinflammation, oxidative and nitrosative damage, microvascular loss, axonal degeneration, and cognitive decline. These phenotypes coincide with a shift in brain EC chromatin accessibility and transcription toward stress-, immune-, and senescence-related programs that mirror the aged EC transcriptome.

Mechanistically, EC KLF4 loss upregulates inflammatory and oxidative stress genes while downregulating pathways required for BBB maintenance. This transcriptional reprogramming likely impairs barrier and vasoregulatory functions, promoting a feed-forward cycle of microglial activation and free radical-mediated neuronal injury. Integration of ATAC-seq, Pol II profiling, ChIP-seq, and RNA-seq links altered chromatin accessibility to active transcriptional changes, supporting a model in which KLF4 preserves endothelial homeostasis by repressing proinflammatory and senescent programs at the chromatin level.

These results extend the known protective roles of KLF family members in large-vessel endothelium to the microvasculature. Prior work showed that KLF2 and KLF4 support anti-inflammatory, anticoagulant endothelial phenotypes and protect against large vessel disease (atherosclerosis, stroke) (17, 70, 71). Combined endothelial loss of KLF2/KLF4 causes catastrophic microvascular failure (26). Here, we observed that single gene loss of EC KLF4 produces a subtler, progressive phenotype of accelerated vascular aging and dementia-relevant pathology. Given the contribution of microvascular dysfunction to systemic age-related disorders (kidney disease, heart failure, sarcopenia, ocular disease), preserving EC KLF4 may have therapeutic value beyond neurodegeneration.

We used an inducible, EC-targeted Cre line, so peripheral EC dysfunction may have contributed to the phenotype. Future studies using brain region-specific manipulations or targeted KLF4 reconstitution will clarify central versus peripheral contributions. Our findings align with recent human vascular single cell data that nominate KLF4, EP300, and MECOM as upstream regulators of Alzheimer’s-associated vascular gene programs (72). Notably, KLF4 and EP300 interact and have been implicated in aging (16, 73), suggesting a possible KLF4-EP300 cassette that governs vascular aging.

Vascular impairment is increasingly implicated in dementia, with current estimates suggesting that 15-30% of dementia cases are primarily vascular. We previously showed that restoring BBB integrity can restore brain health, halt axonal degeneration, and reverse cognitive deficits one year after TBI in mice (64), indicating the disease-modifying potential of EC-directed interventions. Additionally, KLF4 activity may also be modulated by gasotransmitters, such as nitric oxide (NO) and hydrogen sulfide (H_2_S), which act through S-nitrosylation (74–76) and S-sulfhydration (77–80), respectively. S-nitrosylation impairs KLF4 function (81), whereas S-sulfhydration appears protective (82), suggesting therapeutic opportunities to modulate KLF4 via posttranslational pathways.

In summary, EC KLF4 loss is sufficient to reproduce aging-like vascular and cognitive pathology, positioning KLF4 as a compelling therapeutic target to preserve microvascular health and mitigate age-associated cognitive decline. Further exploration of KLF4-centered strategies, including small molecules, posttranslational modulators, or gene-therapy, warrants investigation.

## Materials and Methods

Confirmation of brain endothelial cell–specific KLF4 elimination in mice, study design, details on animals used, behavioral analyses (rotarod, open field, light/dark box, Morris water maze, Barnes maze, forelimb grip strength), two-photon microscopy assessment of neurovascular function (cranial window preparation, arteriolar diameter and capillary blood flow measurement and analysis, BBB permeability measurement and analysis), biochemical dextran extravasation assay, BBB deterioration analysis using transmission electron microscopy, histological assays (immunohistochemistry, image acquisition and analysis, oxidative and nitrosative damage staining and analysis), western blot analysis, open chromatin structure from brain ECs by ATAC-seq and data analysis, RNA Pol II ChIP-seq and data analysis, ATAC-seq and scRNAseq data integration, ChIPseq and ATAC-seq data integration, and statistical analysis details are available in *SI Appendix*.

## Supplementary Material

Appendix 01 (PDF)

Dataset S01 (XLSX)

## Data Availability

All sequencing data generated in this study have been deposited in the NCBI Gene Expression Omnibus (GEO) under accession numbers GSE333394 (scRNA-seq) (83), GSE333395 (ATAC-seq) (84), and GSE333397 (RNA Pol II ChIP-seq) (85). All other study data are included in the article and/or supporting information.
