# Supplementary material for: Endothelial KLF4 depletion drives age-related neurovascular dysfunction and neuropsychiatric impairment
Source: Proc Natl Acad Sci U S A. 2026 Jun 18;123(25):e2426990123. doi: 10.1073/pnas.2426990123 (PMC13291589; doi:10.1073/pnas.2426990123)
Supplement: Supplementary file 1 — Appendix 01 (PDF) [file pnas.2426990123.sapp.pdf]

Supporting Information for

Endothelial KLF4 depletion drives age-related neurovascular dysfunction and neuropsychiatric impairment

Matasha Dhar[a,b,c,d,1], Edwin Vázquez-Rosa[a,b,c,d,1], Kalyani Chaubey[a,b,c,d], Emiko Miller[a,b,c,d,f], Sofia G. Corella[a,b,c,d], Suwarna Chakraborty[e], Sunil Jamuna Tripathi[e], Tapatee Das[g], Xudong Liao[g], Mohamed Alkassem Alosman[g], Hua Fang[a,b,c,d,h,i], Yeojung Koh[a,b,c,d,j], Preethy S. Sridharan[a,b,c,d,f], Kathryn Franke[a,b,c,d], Coral J. Cintrón-Pérez[a,b,c,d], Adrian A. Cintrón-Pérez[a,b,c,d], Taylor Tomco[a,b,c,d], Vidya Indrakumar[a,b,c,d], Phoebe Rubin[a,b,c,d,k], Justin G. Pieper[a,b,c,d,l], Luke A. Ashiku[a,b,c,d,m], Min-Kyoo Shin[a,b,c,d,n], Xinmiao Tang[o], Roshan Padmanabhan[p], Hariprakash Haragopal[q], Margaret E. Flanagan[r,s,t], Rajan Jain[u], Bradley D. Winters[q], Brigid M. Wilson[c,v], Bindu D. Paul[e,w,x,y], Mukesh K. Jain[z,*], Andrew A. Pieper[a,b,c,d,f,j,*]

[a]Department of Psychiatry, Case Western Reserve University, Cleveland, OH, USA

[b]Brain Health Medicines Center, Harrington Discovery Institute, University Hospitals Cleveland Medical Center, Cleveland, OH, USA

[c]Geriatric Psychiatry, GRECC, Louis Stokes VA Medical Center, Cleveland, OH, USA

[d]Institute for Transformative Molecular Medicine, School of Medicine, Case Western Reserve University, Cleveland, OH, USA

[e]Department of Physiology, Pharmacology & Therapeutics, Johns Hopkins University School of Medicine, Baltimore, MD, USA

[f]Department of Neurosciences, Case Western Reserve University, Cleveland, OH, USA

[g]Department of Molecular Biology, Cell Biology & Biochemistry, Division of Biology and Medicine, Brown University, Providence, RI, USA

[h]Hathaway Brown School, Shaker Heights, OH, USA

[i]Massachusetts Institute of Technology; Cambridge, MA, USA

[j]Department of Pathology, Case Western Reserve University, Cleveland, OH, USA

[k]University of California Santa Barbara, Santa Barbara, CA, USA

[l]Northeastern University, Boston, MA, USA

[m]Cate School, Carpinteria, CA, USA

[n]College of Pharmacy and Research Institute of Pharmaceutical Sciences, Seoul National University, Seoul, 08226, Republic of Korea

[o]Yueyang Hospital, Shanghai University of Traditional Chinese Medicine, Shanghai, China

[p]Trailhead Biosystems Inc., Beechwood, OH, USA

[q]Department of Anatomy and Neurobiology, Northeast Ohio Medical University, Rootstown, OH, USA

[r]Glenn Bigg's Institute for Alzheimer's & Neurodegenerative Diseases, University of Texas Health Science Center at San Antonio, San Antonio, TX, USA

[s]Department of Pathology, UT Health Science Center at San Antonio, San Antonio, TX, USA

[t]Mesulam Center for Cognitive Neurology and Alzheimer's Disease, Feinberg School of Medicine, Northwestern University, Chicago, IL, USA

[u]Departments of Medicine and Cell and Developmental Biology, Penn Cardiovascular Institute, Penn Epigenetics Institute, Perelman School of Medicine, University of Pennsylvania, Philadelphia, PA, USA

[v]Division of Infectious Diseases and HIV Medicine in the Department of Medicine, Case Western Reserve University, Cleveland, OH, USA
[w]Department of Psychiatry and Behavioral Sciences, Johns Hopkins University School of Medicine, Baltimore, MD, USA
[x]The Solomon H. Snyder Department of Neuroscience, Johns Hopkins University School of Medicine, Baltimore, MD, USA
[y]Lieber Institute for Brain Development, Baltimore, MD, USA
[z]Department of Molecular Biology, Cell Biology and Biochemistry, The Warren Alpert Medical School, Brown University, Providence, RI, USA

[1]These authors contributed equally to this work.
*Correspondence: mukesh_jain@brown.edu (M.K.J.) or andrew.pieper@case.edu (A.A.P.)

This PDF file includes:

Supporting text (Methods)

Figures S1 to S6

SI References

Methods

Confirmation of brain endothelial cell-specific KLF4 elimination in mice

Brain tissue was collected from adult mice after seven days of tamoxifen injection (2 mg/25 g), in accordance with institutional animal care and use guidelines. Dissected brains were minced and digested using an enzyme cocktail containing Liberase, hyaluronidase, and DNase to promote efficient dissociation. Mechanical processing was conducted with a gentleMACS dissociator (Miltenyi Biotec). Myelin and debris were removed to generate a purified single-cell suspension. Endothelial cells were enriched using CD31-conjugated magnetic microbeads (Miltenyi Biotec) following the manufacturer's recommendations. Magnetically retained $CD31^+$ cells were collected as the endothelial fraction and used immediately for downstream molecular assays. Purified endothelial cells were processed immediately for RNA and protein analysis. Total RNA was isolated using standard column-based extraction methods and quantified prior to cDNA synthesis. Gene expression was measured by quantitative PCR using validated primer sets and relative expression levels were determined using established normalization methods. For protein analysis, endothelial cell lysates were prepared using detergent-based extraction buffers. Equal

amounts of protein were separated by SDS–PAGE and transferred to membranes for immunoblotting. Membranes were probed with antibodies against endothelial markers and loading controls, followed by detection using standard chemiluminescent or fluorescence-based methods.

Animals

Animal studies were conduted in accordance with animal care guidelines from the Institutional Animal Care Use Committee (IACUC) at Case Western Reserve University. Animal behavior studies were conducted at the Louis Stokes Cleveland VA Medical Center and approved by Louis Stokes Cleveland VA Medical Center Institutional Animal Care and Use Committee. Male and female endothelial-specific KLF4 knockout (VE-Cadherin Cre x Floxed KLF4) and VE-Cadherin Cre controls were used at specified ages. This mouse line is on the C57BL/6J background. To trigger endothelial-specific deletion of *Klf4* gene, 8 week-old mice were intraperitoneally injected with tamoxifen (100ul for 5 days) (MP Biomedicals). The animal housing facility is pathogen-free, with temperature and humidity controlled. Mice had ad libitum access to water and regular laboratory rodent chow and were exposed to a 12-hour light/dark cycle. Exact p values for each panel in each figure are included in Dataset S1.

Behavioral analysis

Rotarod test: This test was conducted on RotaMax5 from Columbus instruments over 3 days. Day 1 started with a 1-minute habituation session on stationary rotarods followed by three 5-minute trials on non-accelerating rotarods rotating at 5 rpm. Day 2 consisted of three 5-minute trials on non-accelerating rotarods rotating at 5 rpm. Day 3 consisted of three 5-minute trials on accelerating rotarods, starting at 3 rpm and increasing in rotation speed every 20 seconds. Latency to fall from the accelerating rotarods was automatically measured by beam brake on RotaMax5.

Open field test: This test was conducted in an opaque box (40x40x35cm^3, Stoelting Co.) placed on a raised surface (3-4 foot off the floor) with the camera mounted to the ceiling above the apparatus. Mouse behavior was recorded and analyzed using Any-maze video tracking software

(Stoelting Co.). Mice were allowed to explore the open field for 10 minutes. The apparatus was thoroughly cleaned between animals. Center zone was defined as the 10 cm^2 in the center of the field and the border zone was defined as the region 8 cm from the edge of the field. Entry in a zone was defined by forepaws crossing into the zone, while exit from a zone was defined by all four paws being out of the zone. Freezing was detected when a mouse remained in one position without motion detected by the software for 10s or greater.

<u>Light/Dark Box test:</u> This test was conducted in a box (40cm^3, Stoelting Co.) placed on a raised surface (3-4 foot off the floor) with the camera mounted to the ceiling above the apparatus. Half of the box had transparent walls (light zone) while the other half had opaque walls and an opaque top (dark zone). Mouse behavior was recorded and analyzed using Any-maze video tracking software (Stoelting Co.). Mice were allowed to explore the light/dark box for 5 minutes. Entry in a zone was defined by forepaws crossing into the zone, while exit from a zone was defined by all four paws being out of the zone.

<u>Morris Water Maze test:</u> This test was conducted in a 128cm diameter tank filled with 19 cm of room temperature water (22 °C). A transparent submerged (~1.5 cm below water surface) platform (9cm diameter) was used to obscure the location of the platform, which was kept consistent over the four days of training. Four different and equally spaced visual cues with different shapes and colors were placed inside the tank. Each training day consisted of four trials per day (60s per trial) for four consecutive days. If the mouse did not find the platform within 60s it was gently placed on the platform for 20s before being removed from the apparatus. Upon removal, all mice were dried with paper towels and placed in the home cage. Inter-trial intervals were 30 minutes and the postion of where the mouse was placed in the apparatus was changed for an animal per training day. During probe test on day 5, the platform was removed and each animal was monitored for 30s. Any-maze video tracking software (Stoelting Co.) was used to measure latency to find the hidden platform, platform crossing, time in target quadrant, and mean speed.

<u>Barnes Maze test:</u> This test was conducted on a gray circular platform (91 cm in diameter and 90 cm in height), with 20 equally spaced holes 5 cm in diameter along the perimeter (Stoelting Co.). One of these holes contained a recessed escape chamber located under the platform. Four different and equally-spaced visual cues with different shapes and colors were hung on a black circular curtain surrounding the maze. The training session consisted of four trials per day for four

consecutive days. In each trial, the mouse was gently released in the middle of the maze under the cylindrical chamber, and after 5s elapsed the covering chamber was lifted to allow the mouse to explore the maze. If a mouse failed to find the escape chamber within 120s, it was manually guided to the escape chamber and then allowed to stay in the chamber for 30s. Both the platform and the escape chamber were cleaned thoroughly between individual trials. On day 5, 24h after the last training day, the escape chamber was removed and mice were allowed to explore the maze for 120s. Total and primary latency were measured during training days, and latency to first nose pokes in escape hole and time spent in target quadrant were measured for memory test on the probe trial day. Any-maze video tracking software (Stoelting Co.) was used to acquire measurements.

Forelimb grip strength test: This test was conducted using a force meter attached to a small wire hanger. Mice were held by the base of the tail and allowed to grasp the wire hanger. A gentle tug to the tail nudges the mice to pull on the wire and the force generated was recorded as grip strength. Three trials were conducted per mouse on the same day.

Two-photon microscopy assessment of neurovascular function

Cranial window preparation: Mice were deeply anesthetized using isoflurane and placed in a stereotaxic frame with continued anesthetic support. The surgical area above the skull was prepared using standard pre-operative procedures. The surgical site was numbed using 100ul of 1% lidocaine applied above the skin. Skin, fascia, and muscle above the craniotomy location were removed. Craniotomy was performed using a 0.5 mm drill bit attached to a dental drill. Care was taken to not put excessive pressure while drilling and the site was repeatedly washed with sterile saline to avoid overheating and to remove bone fragments. A 3 mm^2 portion of the skull centered above hindlimb somatosensory cortex (AP= -0.95mm, L= 1.5mm) was removed gently. The craniotomy was sealed with a sterile glass optical window pre-cut to fit just inside the craniotomy. The window was sealed to the skull using veterinary glue. The skull and the edge of the skin around the craniotomy were sealed with dental cement. An aluminum head plate (Narishige CF10 or CP-2) was glued to the dental cement. After surgery, animals were given a 5mg/kg dose of meloxicam subcutaneously and all standard post-operative care procedures were followed. Animals were allowed to recover for minimum of 2 weeks post-surgery before imaging.

Arteriolar diameter and capillary blood flow measurement and analysis: To acquire baseline and stimulus induced changes in neurovascular function, the method described in Kisler et al. (1) was followed. All imaging was performed under isoflurane anesthesia (1-2%). Animals were injected with 100ul of 70kD FITC -dextran (10mg/ml) retro-orbitally to label the blood plasma, allowing for imaging of the vasculature through the cranial window. Imaging was performed using an Olympus FVMPE-RS Multiphoton Laser Scanning microscope with a mode-locked Ti:sapphire laser (Insight X3 Dual with Deep See; Spectra Physics) set to 920nm and emission for FITC was collected by a 495-550 nm bandpass filter. Arteries were identified by their morphology, speed, and direction of blood flow. Arterioles emerging from these arteries with a diameter between 10-40μm and capillaries emerging from the arterioles with dimeter less than 6μm were targeted for neurovascular imaging. For changes in arteriolar diameter, line scan perpendicular to the arteriole were acquired at the rate of 10Hz and 0.09um/pixel resolution for the duration of 25s per vessel, with first 5s as baseline followed by 10s stimulation and then 10s of post-stimulus imaging. Imaging and stimulus delivery were coordinated by Digidata 1550 and pClamp software controlling a pulse-generator and microscope I/O box (FV-30 analog). Acquired images were converted to binary images using ImageJ thresholding and analyzed using a custom MATLAB script which counted the number of white pixels per line and converted it into diameter measurement per line. The diameter vs time data generated was further filtered via 1Hz low-pass filter, 0.5Hz notch filtered, and 1s sliding box filter within the MATLAB script to remove noise and breathing artifacts. For changes in capillary blood flow, line scan parallel to the blood flow within the vessel was acquired at the rate of 800Hz and 0.09um/pixel resolution for the duration of 25s per vessel, with first 5s as baseline followed by 10s stimulation and then 10s of post-stimulus imaging. Acquired images were analyzed using previously published MATLAB script (2) to generate velocity vs time data which was further filtered via 1Hz low-pass filter, 0.5Hz notch filtered, and 1s sliding box filter we added to the previously published MATLAB script to remove noise and breathing artifacts. Hindlimb stimulation was performed by delivering a mild electrical pulse (10s, 10Hz, 2ms pulse duration) below the contralateral hind paw skin using fine subdermal needles (Spes medica LLC).

BBB permeability measurement and analysis: BBB permeability was measured by the method of Janiurek et al. (3). All imaging was performed under isoflurane anesthesia (1-2%). Animals were injected with 100ul of 3kD FITC -dextran (10mg/ml) retro-orbitally and immediately head fixed under the microscope for imaging. Four fields of view (FOV) (500μm^2, 0.9μm/pixel) under the cranial window were selected for imaging and 100μm z-stack were acquired every 2 minutes from

each FOV over a 30 minute duration. Analysis was performed on 2D projection images using ImageJ. 20 ROIs within the vessels and 20 ROIs within the parenchyma were chosen per image to generate fluorescence intensity over time plots. Fluorescence intensity from the first image in the timeseries was used as baseline to generate change in fluorescence overtime data.

Biochemical dextran extravasation assay: For biochemical assessment of BBB permeability, mice were anesthetized with ketamine (100mg/kg) and xylazine (10mg/kg). After confirmation of anesthetic depth with toe pinch, mice were injected with 100ul of 3-kDa TMR dextran (1mg/ml, D3308; Invitrogen) retro-orbitally. Blood was then collected by cardiac draw 5 min after dextran injection and perfused with phosphate-buffered saline (PBS) for 5 min. After perfusion, brain tissue was collected and frozen for storage at -80ºC. All tissue processing was conducted with protection from light to main TMR fluorescence. Brain was homogenized in 600ul PBS and fluorescent intensity was measured in homogenate using a Spectramax M2 microplate reader and normalized to fluorescent intensity from plasma samples collected from the same animals.

BBB deterioration analysis using transmission electron microscopy: Anesthetized mice were transcardially perfused with a fixative solution (1/4-strength Karnovsky's fixative) at a 10 ml/min flow rate for ten minutes. Brain tissues were fixed with 2.5% glutaraldehyde and 2% paraformaldehyde in 0.1 M HEPES buffer, pH 7.4 for two hours at room temperature. The brain tissue underwent a thorough rinse in 0.1 M phosphate buffer at pH 7.4, followed by a two-hour postfixation in an unbuffered 1:1 mixture of 2% osmium tetroxide and 3% potassium ferricyanide. After a rinse with distilled water, the brain tissue was immersed overnight in an acidified 0.25% uranyl acetate solution. Following another rinse with distilled water, the tissue underwent dehydration in increasing ethanol concentrations, was treated with propylene oxide, and was embedded in a mixture of EMbed 812 resin (Electron Microscopy Sciences, PA). Thin sections measuring 70 nm were sliced using an RMC MT6000-XL ultramicrotome and placed onto Gilder square 300 mesh nickel grids (Electron Microscopy Sciences, PA). Sections underwent staining with acidified uranyl acetate, followed by a modified version of Sato's triple lead stain. These were visualized with a FEI Tecnai Spirit (T12) equipped with a Gatan US4000 4kx4k CCD.

Histological assays

Immunohistochemistry: Mice were anesthetized with ketamine (100mg/kg) and xylazine (10mg/kg). After confirmation of anesthetic depth with toe pinch, transcardiac perfusion was performed with cold PBS followed by 4% paraformaldehyde in PBS at pH 7.4. Brains were carefully removed and post-fixed in 4% paraformaldehyde for 24h followed by submergence in 30% sucrose in PBS for 48-72h at 4ºC. Submerged brains were rapidly frozen in 2-methylbutane pre-cooled to -20ºC with dry ice. Frozen brains were cut on a microtome at 40µm thickness coronally and sections were stored in cryoprotective solution (150mM Ethylene glycol, 100mM glycerol, 250mM PBS) at - 20ºC. For immunohistochemistry, sections were washed in PBS three-times for 5 min each. Blocking and permeabilization was performed simultaneously in 5% Normal donkey serum (NDS) and 5% bovine serum albumin (BSA) in 0.25% TritonX-100 in PBS (PBST) for 2h at room temperature. Sections were incubated in primary antibodies made in 1% NDA and 1%BSA in PBST for 1-2d at 4ºC. After washing in PBS (3 times, 5min each), sections were incubated in appropriate mix of secondary antibodies made in 1% NDA and 1%BSA in PBST for 2h at room temperature. After washing in PBS, sections were mounted and coverslip in DAPI-fluoromount-G (Southern biotech). The primary antibodies and dilutions used were as follows: anti-fibrin (1:500, PA5-95397, Invitrogen), anti-Iba1 (1:500, 019-19741; Fujifilm Wako); anti-CD-68 (1:500, MCA1957GA, BioRad); anti-CD31 (1:100, 550274; BD Biosciences); anti-GADD34 (1:500, 10449-1-AP, Proteintech); anti-ADAMTS1 (1:500, 12749-1-AP, Proteintech), anti-MCT1 (1:500, 20139-1-AP, Proteintech), and anti-CD13 (1:200, AF2335, R&D systems). For IgG staining, anti-mouse Cy3 IgG was used with the secondary antibodies. For lectin staining, FITC-conjugated Lectin (1:500, FL-1171, Vector Labs) was used with the secondary antibodies. The secondary antibodies and dilutions used were as follows: anti-rat Alexa 488 (1:1,000, A32723; Invitrogen); anti-rabbit Alexa 488 (1:1,000, A21206; Invitrogen); and anti-goat Alexa 647(1:1,000, A21447; Invitrogen). For silver staining, free-floating brain sections were processed and stained with an FD NeuroSilver Kit (FD NeuroTechnologies, Columbia, MD) and quantified per our standard procedure (PMID: 25220467, 27822499, 30421909, 33087571, 33852912, 39241772).

Image Acquisition and Analysis: Images were acquired using the Zeiss Axio Imager.M2 microscope and Zeiss Axio Scan.Z1, keeping the light intensity and exposure time constant. ImageJ version 1.42 software (NIH, Bethesda, MD) was used to analyze brightfield and fluorescent images. IgG and Fibrin staining analysis, signal from the Lectin (vessel staining) channel was used to generate a vessel mask which was subtracted from the IgG/fibrin channel

to generate a parenchymal-only measure of IgG/fibrin fluorescence intensity. For IBA1 area analysis, images were converted to binary image using the ImageJ threshold plugin and area of particles was measured using Analyze particles plugin. CD68 area was measured in IBA+ cell mask, using Analyze particles plugin. Vessel length measurement was performed by Simple Neurite Tracer for CD31 positive capillaries. CD13 positive pericytes were counted and pericyte coverage was calculated by the ratio of number of pericytes divided by capillary length. Silver staining (black staining) was quantified using the plugin of the color deconvolution method described by Ruifrok and Johnston (4).

<u>Oxidative and nitrosative damage staining and analysis:</u> Sections were rinsed with phosphate-buffered saline (PBS) containing 0.3% Triton X-100 (TX) and then blocked with 5% normal goat serum. Following blocking, sections were incubated overnight at 4 °C either with anti-4-hydroxynonenal (1:500, Rabbit Polyclonal, Alpha Diagnostic International, Cat# HNE11-S) or anti-3-Nitrotyrosine (1:500, Rabbit Polyclonal, Millipore Sigma, Cat# AB5411). The next day, sections were washed with PBS-TX and incubated for two hours at room temperature (RT) with appropriate secondary antibodies conjugated with Alexa 594 or 488 (1:300; Invitrogen). Finally, the sections were rinsed in PBS and mounted using antifade aqueous media (Vectashield® Plus with DAPI, Vector Laboratories). Images were captured on LSM 710, and mean fluorescence intensities were quantified using Fiji ImageJ (National Institutes of Health, Bethesda, MD).

Western Blot Analysis: Brain cortex tissues were homogenized in RIPA buffer (R0278; Sigma-Aldrich) supplemented with protease and phosphatase inhibitor cocktail (1861284; Thermo Scientific). Homogenates were centrifuged at 14,000 rpm at 4 °C for 30 min, after which supernatants were collected, and protein concentration was measured by BCA Protein Assay Kit (A53225; Thermo Scientific). Proteins were resolved in 4-20% Criterion TGX Stain-Free gels (5678095; Bio-Rad Laboratories) and transferred onto 0.2 µm PVDF membranes (1704157; Bio-Rad Laboratories). Membranes were blocked with 5% nonfat dry milk in tris-buffered saline-tween 20 (TBST) for 1 h at room temperature and incubated with primary antibodies at 4°C overnight. The following primary antibodies were used: anti-Claudin 5 (1:500, 34-1600, Thermo Fisher Scientific); anti-ZO-1 (1:500, 33-9100, Thermo Fisher Scientific); anti-Occludin (1:500, 701161, Thermo Fisher Scientific), and anti-GAPDH (1:2000, MAB 374, EMD Millipore). After primary antibody incubation, membranes were rinsed with TBST (3 x 5 min) and then incubated with HRP-conjugated secondary antibodies. SuperSignalTM West Femto Maximum Sensitivity Substrate

(34096, Thermo Scientific) was used for developing and image acquisition using Bio-Rad ChemiDoc Imaging System. Densitometry analysis was conducted using Bio-Rad Image Lab Software.

Open Chromatin structure from Brain ECs by ATAC-seq and data analysis: A total of 80,000 to 90,000 flow-sorted live brain ECs were used to prepare the ATAC-seq library. The OMNI ATAC-seq protocol (5) was adopted for isolating nuclei and applying transposase reaction followed by 12 cycles of polymerase chain reaction (PCR) amplification (6). The library was purified using two-sided SPRI beads selection (100-500 bp fragment size). The Illumina Hi-Seq system was used to sequence libraries. The reads from raw files were trimmed from Illuminia adapter using TrimGalore (v.0.5.0) and mapped to the mouse genome (mm10 version) using Bowtie2 (v.2.3.0) (PMID: 22388286) with specific options (--local , --very-sensitive-local, -- phred33, -N 1). To filter the mapped reads, Picard tools (v. 2.9.2) and SAMtools (v. 1.9) (7) were employed. Unmapped reads, failed primary alignments, and duplicates were removed, while properly paired reads with MAPQ >30 were retained. Subsequently, peaks for open chromatin regions were called using MACS2 (v. 2.2.6) (88) with the parameters -f BAMPE, -g mm and –broad flag. The BAM files and peak bed files were then utilized in DiffBind (v. 3.17). DiffBind: differential binding analysis of ChIP-Seq peak data.) to define differential accessibility regions (DARs) by applying specific functions for read counting (dba.count), library size normalization (dba.normalize), enrichment heatmaps generation (dba.plotprofile), and calling DARs using the DESeq2 method (dba.contrast). Sites showing statistically significant differences between conditions were defined as DARs using an absolute cutoff of fold change (FC) >0.5 and false discovery rate (FDR) <0.05, with non-DARs defined using an FDR >0.05 for further analysis. Motif enrichment searches were performed using Homer (v. 4.11). Using HOMER software, NREL's Micropower Optimization Model, to Explore the Role of Gen-Sets in Small Solar Power Systems; Case Study: Sri Lanka (No: NREL/TP-710-36774). National Renewable Energy Lab to identify DAR peaks. The DeepTools (v. 3.1.0) (9) was employed with bamCoverage function to create coverage Bigwig files, which were then uploaded to IzG (90) for visualization as genomic tracks. Visualizations and statistical tests were

conducted using R/4.2.0. Specifically, peak annotation to genomic regions was performed using the Chipseeker package (11,12) and gene ontology enrichment analysis was carried out using the cluster Profiler package (13) and ReactomePA package for pathway enrichment analysis (14). Boxplots and violin plots were generated using the ggplot2 package. The DeepTools (v. 3.1.0) was employed with bam Coverage function to create coverage Bigwig files, which were then uploaded to IGV for visualization as genomic tracks. Visualizations and statistical tests were conducted using R/4.2.0 (R_Core_Team (2021). R: A language and environment for statistical computing. R Foundation for Statistical Computing, Vienna, Austria). Specifically, peak annotation to genomic regions was performed using the Chipseeker package (11,12) and gene ontology enrichment analysis was carried out using the clusterProfiler package (13) and ReactomePA package for the pathway enrichment analysis (14). Boxplots and violin plots were generated using the ggplot2 package (ggplot2: Elegant Graphics for Data Analysis).

RNA Pol II ChIP-seq and data analysis: Flow-sorted endothelial cells (200,000) were fixed using 1% formaldehyde at room temperature for 10 minutes with swirling and then quenched with 0.125M glycine for 5 min at room temperature. Cross-linked cells were lysed and frozen in 100 µL SDS lysis buffer (50 mmol/L Tris/HCl, 0.5% SDS, and 10 mmol/L EDTA) supplemented with 1x complete EDTA-free protease inhibitor**.** Cells were rinsed twice with cold PBS before pelleting and stored until MNase digestion. For MNase digestion, the cell pellet was resuspended in PBS with 0.1% Triton X-100 supplemented with 1 mM $CaCl_2$. 100 U of MNase (NEB) were added to pre-warmed cells and incubated at 37 °C for 15 min. Digestion was halted by moving to ice and adding 10 µl of 250 mM EDTA. Before DNA clean-up, digestions were adjusted to 0.5% SDS and 10 mM Tris pH 8. For DNA cleanup, digestions were incubated with RNase (Roche) for 30 min at 37 °C, with proteinase K (Roche) for 60 min at 55 °C, and then incubated at 65 °C for 60 min to reverse crosslinks. After MNase digestion, DNA fragment size was checked with desirable DNA fragment size (200-800bp). For chromatin immunoprecipitation followed by sequencing 5ug of ChIP grade antibody anti Pol-II (Sigma, 05–623) were used, blocked with BSA and cross-linked on magnetic protein A/G based on the antibody of origin, at 4 °C for 4hr. The MNase digested lysate was checked and incubated with the antibody at 4 °C overnight. The ChIP complex was pulled down using a magnetic separator and washed with high sucrose buffer, high salt buffer, LiCl buffer with protease inhibitor, and then with a final Tris wash. The immune complex was eluted with ChIP elution buffer and reverse cross-linked at 65 °C overnight. After reverse

crosslinking, 1 µL 20 µg/mL RNase (Sigma) was added and incubated at 37 °C for 30 minutes. DNA purification was carried out using the Zymo PCR Purification Kit. 15 µL of PCR master mix and 5 µL of primer mix (Nextera, Illumina) were added to 20 µL of eluted DNA and libraries were amplified. After PCR amplification, library cleanup was done using Agencourt AmPureXP beads (Beckman Coulter) at a ratio of 1:1. DNA concentrations in purified samples were measured using the Qubit dsRNA HS Kit (Invitrogen). Libraries were pooled and paired-end sequenced using the NextSeq 500 platform (Illumina).

To analyze our ChIP-seq data, we used TrimGalore/0.5.0 to trim the reads and remove Illumina adaptors. The trimming process involved applying a minimal phred score of 30 and discarding reads below a minimal length of 36 bp. Next, we employed Bowtie2 (v. 2.3.0) with the alignment options followed in ATAC-seq to map the cleaned reads to the mouse genome (mm10 version). PCR duplicates were removed using Picard tools (v. 2.9.2) and SAMtools (v. 1.9) (7). We then utilized MACS2 (v. 2.2.6) (8) to identify peaks with default parameters, and MSPC (v. 4.0.0) (15) to generate consensus peaks from the biological replicates. To generate enrichment heatmaps and average profiles, we utilized DeepTools (v. 3.1.0) (9). Bigwig files were created using DeepTools bamCompare.

ATAC-seq and scRNAseq data integration: To intersect ATACseq peaks with DEGens lists, fwe annotated genes near peaks regions in R using the annotatePeak function from the ChIPseeker package (11) and plotted all annotated peaks as the log2FC of DEGenes. This allowed us to define two types of DEGene-related ATAC-peaks based on the intersection results: (1) DEGenes that overlapped with DAR peaks and (2) DEGenes that overlapped with nonDAR peaks.

ChIPseq and ATAC-seq data integration: To identify the relationships between peaks in ATAC-seq DARS regions and CHiPseq Pol II peaks, we used annotatePeak function to annotate regions to the nearest genes and then applied the joint function to identify the overlapped genes. Gene ontology and pathway enrichment analysis were performed on shared genes across analysis conditions to identify related biological process and pathways.

Statistical analysis: All statistical analyses were performed using GraphPad Prism, version 9.0.0 (GraphPad Software, Inc.). Details regarding exact statistical tests used, sample sizes, post-hoc comparisons and p-values are mentioned in the figure legends.

Supplementary Figure 1

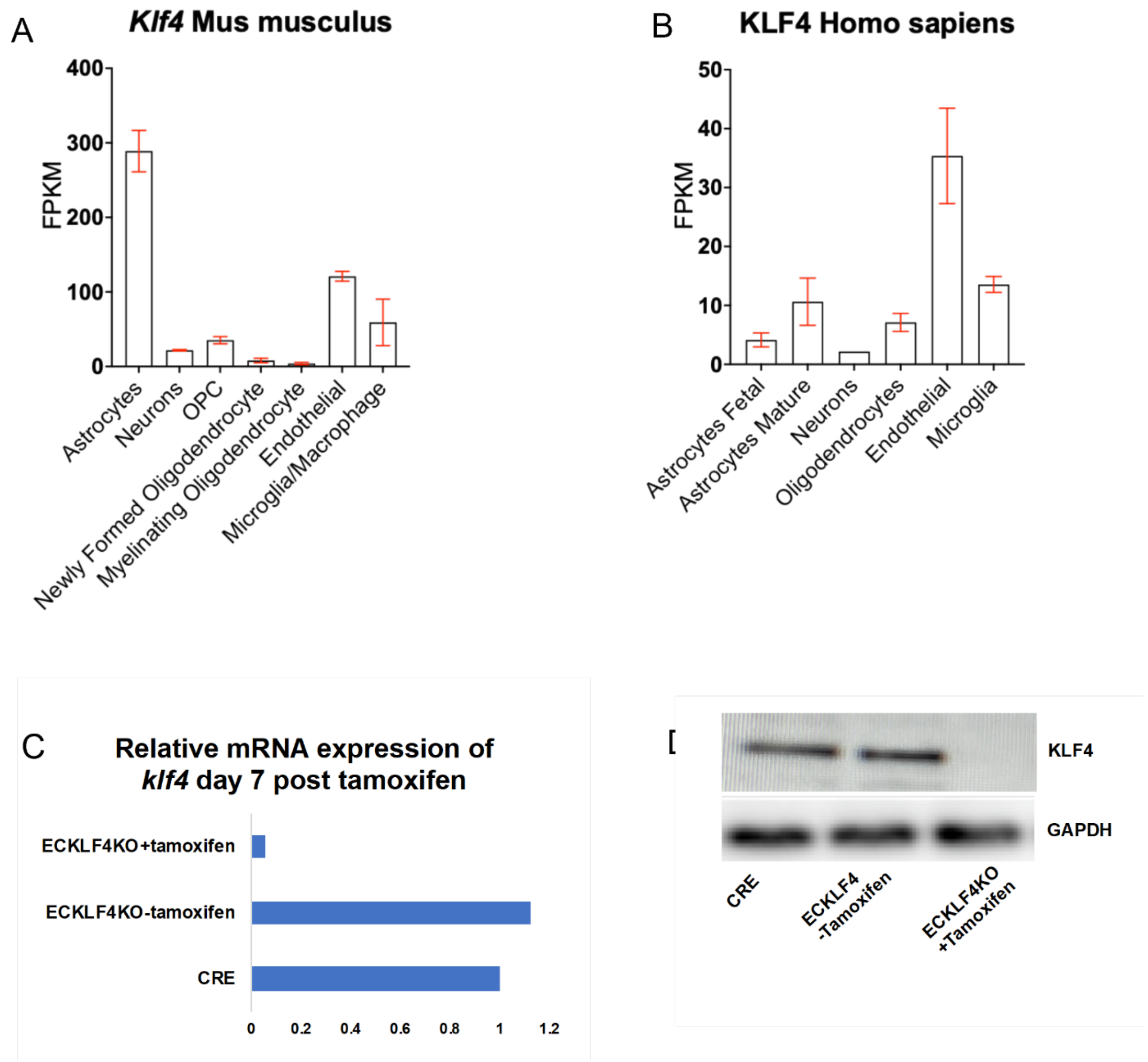

Fig. S1: Distribution of human and mouse KLF4 mRNA in brain cells. RNA sequencing data from Zhang et al. (2014) (22) and Zhang et al. (2016) (23) show KLF4 mRNA distribution among different cell populations in the **a,** mouse and **b,** human brain (OPC = oligodendrocyte precursor cells). **c,** Relative mRNA expression of *klf4* in brain endothelial cells isolated from adult mice post 7days of tamoxifen was normalized with gapdh (n=3). **d,** Representative western blot image for KLF4 protein expression in brain endothelial cells isolated from adult mice after 7days of tamoxifen, normalized with gapdh (n=3).

Supplementary Figure 2

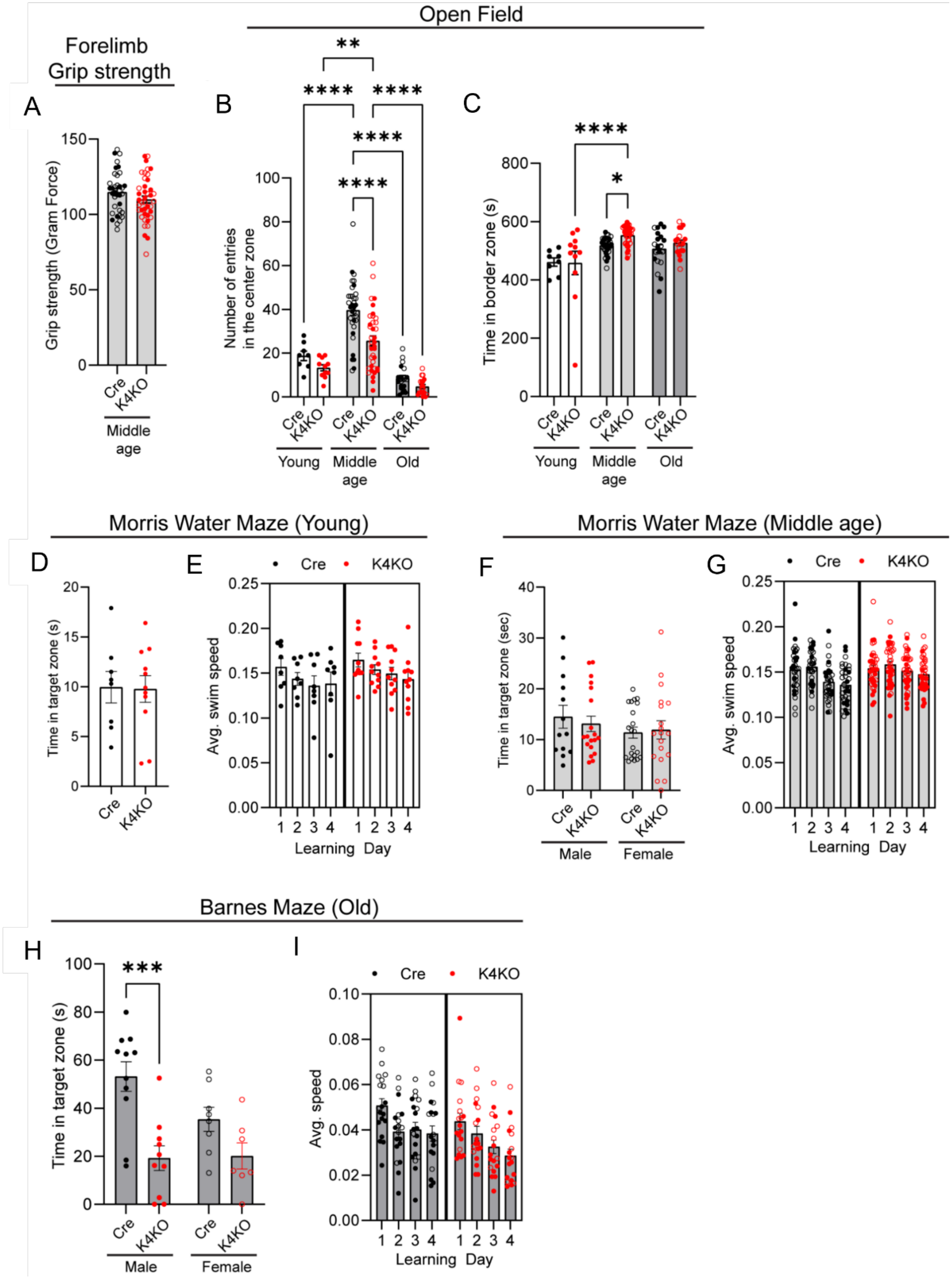

Fig. S2: Control data for experiments showing that loss of endothelial KLF4 accelerates age-associated neurobehavioral and cognitive impairment. a, Forelimb grip strength is equivalent in middle-aged EC-K4KO and WT Cre littermate mice. **b,c,** Anxiety-like behavior tested in the open field shows increased anxiety with age in both WT Cre mice and EC-K4KO mice, as evidenced by reduced entries in the center zone in old WT Cre mice compared to middle-aged WT Cre and in old EC-K4KO mice, compared to middle-aged K4KO mice, although time spent in the border zone was equivalent across groups. EC-K4KO mice also show age-dependent increase in time spent in the border-zone, as well as increased anxiety in middle-aged, compared to middle-aged WT Cre mice. Middle-aged Cre mice enter the center zone more frequently and spend more total time in the border zone than young WT Cre mice, suggesting increased excitability. **d,e,** Time spent in target zone during the Morris water maze probe test and average swim speed over the 4 learning days is the same for young EC-K4KO mice and WT Cre littermates. **f,g,** Time spent in the target zone during the Morris water maze probe test and average swim speed over the 4 learning days is the same for middle-aged EC-K4KO mice and WT Cre littermates. **h,i,** Time spent in the target zone during the Barnes maze probe test was decreased in old EC-K4KO males, compared to WT Cre littermates, while females showed a similar trend. Average speed over the 4 learning days was the same for young EC-K4KO mice and WT Cre littermates. All data shown are average (±SEM). Individual data points represent individual animals with black filled circles as WT Cre males, black open circles as WT Cre females, red closed circles as EC-K4KO males, and red open circles as EC-K4KO females. Significance was tested using one-Way ANOVA and Bonferroni post hoc analysis for all panels. *$p<0.05$, **$p<0.01$, ***$p<0.001$, ****$p<0.0001$

Supplementary Figure 3

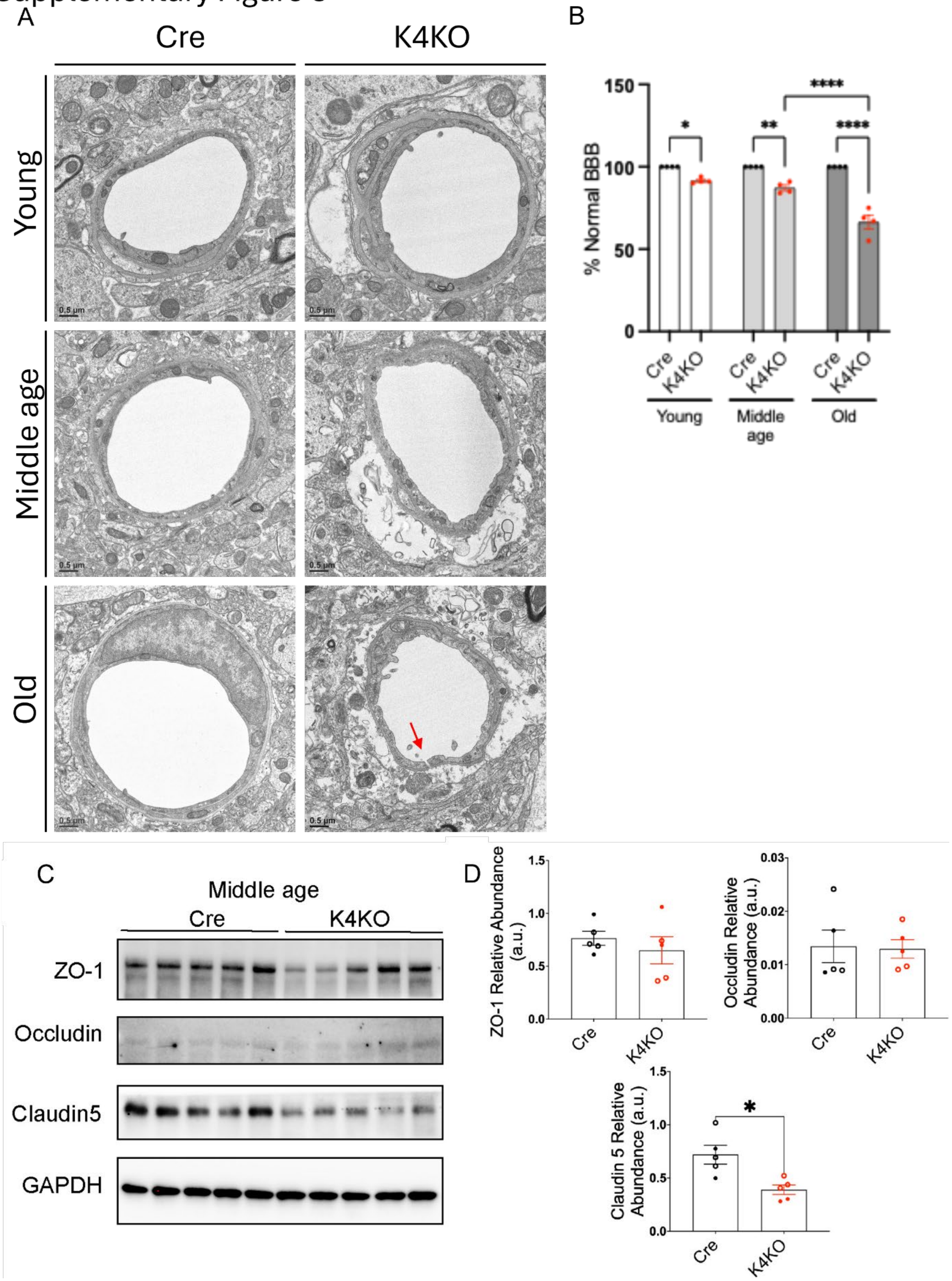

Fig S3: Loss of endothelial KLF4 disrupts the blood-brain barrier (BBB). a, BBB deterioration was measured by transmission electron microscopy. Representative images from EC-K4KO mice and WT Cre littermates demonstrate accumulation of astrocyte endfeet swelling in young, middle-aged, and old EC-K4KO mice, compared with WT Cre littermates. At old age, EC-K4KO mice also show breaks in the capillaries (red arrow). **b.** Quantification of BBB damage demonstrates progressive deterioration in old EC-K4KO mice relative to middle-aged EC-K4KO mice, as well as greater BBB damage in EC-K4KO mice compared to young, middle-aged, and old WT Cre littermate mice. c, Western blot analysis of tight junction proteins in the cortex of middle-aged animals shows reduced levels of Claudin 5 in the EC-K4KO mice compared with WT Cre littermates and no changes in ZO-1 or Occludin. d, Western blot quantification. Data shown are average (±SEM). For the transmission electron microscopy experiment, individual data points represent individual animals. Two males and two females were analyzed in all groups except the old group, in which all animals were male. For tight junction analysis, individual data points represent individual animals with black filled circles as WT Cre males, black open circles as WT Cre females, red closed circles as EC-K4KO males and red open circles as EC-K4KO females. Significance was tested using unpaired t-test (panel D) and one-way ANOVA and Tukey's post hoc analysis (panel B). *p<0.05, **p<0.01, ****p<0.0001.

Supplementary Figure 4

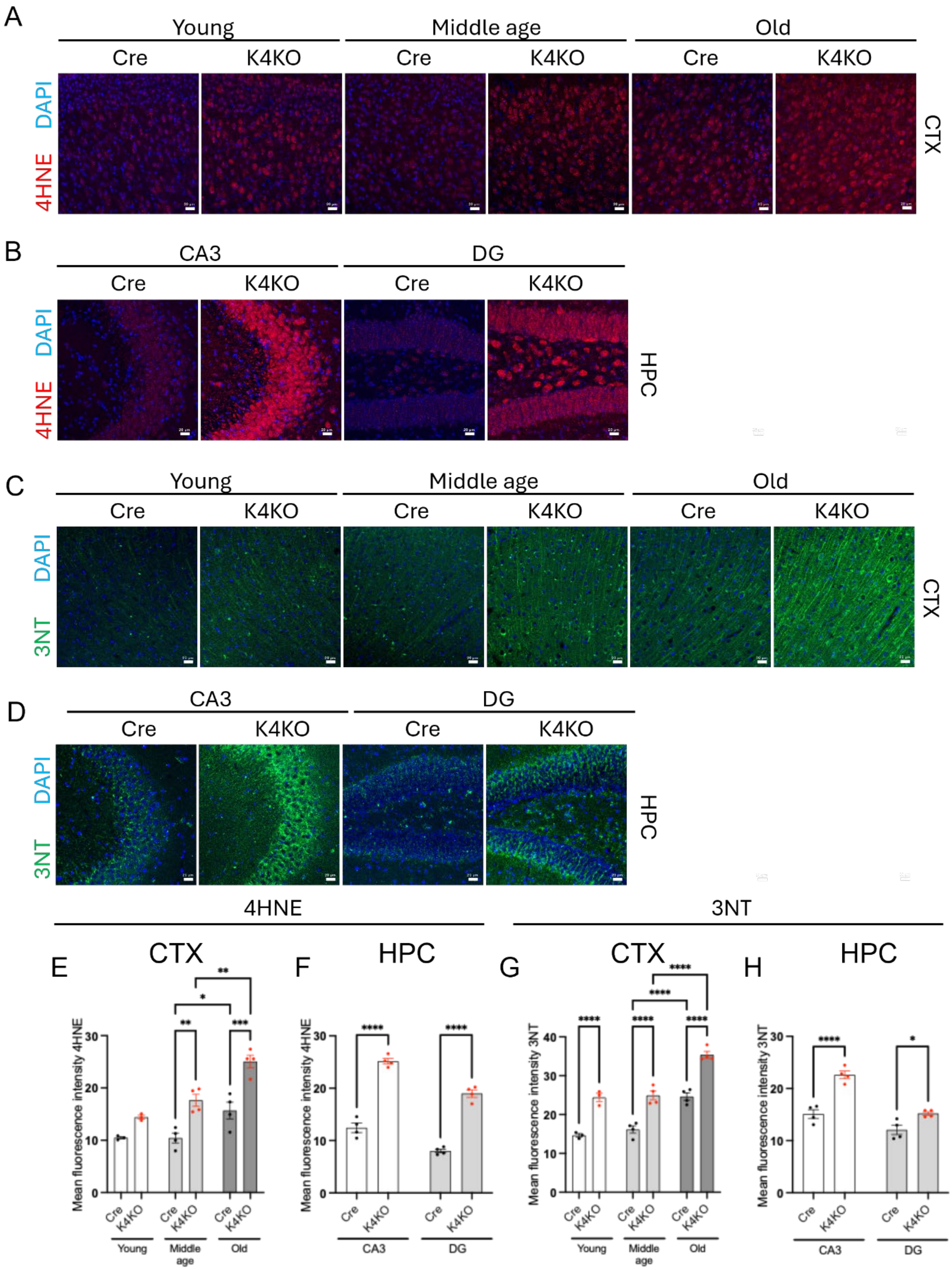

Fig. S4: Loss of endothelial KLF4 elevates oxidative and nitrosative damage in the brain. **a,e,** Representative images and quantification of oxidative damage measured by 4HNE accumulation in the cortex (CTX) of young, middle-aged, and old EC-K4KO and WT Cre littermate mice demonstrate a progressive increase in oxidative damage in aging WT Cre mice that is more pronounced in EC-K4KO mice, as well as greater accumulation of 4HNE in EC-K4KO mice relative to WT Cre littermates in middle-aged and old groups. **b,f.** Representative images and quantification of 4HNE accumulation in the CA3 and dentate gyrus (DG) regions of the hippocampus (HPC) in middle-aged EC-K4KO and WT Cre littermate mice show increased oxidative damage in EC-K4KO mice relative to WT Cre littermates in both regions**. c, g,** Representative images and quantification of nitrosative damage measured by 3NT accumulation in the CTX of young, middle-aged, and old EC-K4KO and WT Cre littermate mice demonstrate a progressive increase in nitrosative damage in aging WT Cre mice that is more prominent in EC-K4KO mice, as well as greater accumulation of 4HNE in EC-K4KO mice, relative to young, middle-aged, and old WT Cre littermates. **d,h** Representative images and quantification of 3NT accumulation in CA3 and DG regions of HPC in middle-aged EC-K4KO and WT Cre littermate mice show increased nitrosative damage in EC-K4KO mice, relative to Cre littermates, in both regions. Data shown are average (±SEM). Individual data points represent individual animals. Significance was tested using one-way ANOVA and Tukey's post hoc analysis for all panels. *$p<0.05$, **$p<0.01$, ***$p<0.001$, ****$p<0.0001$.

Supplementary Figure 5

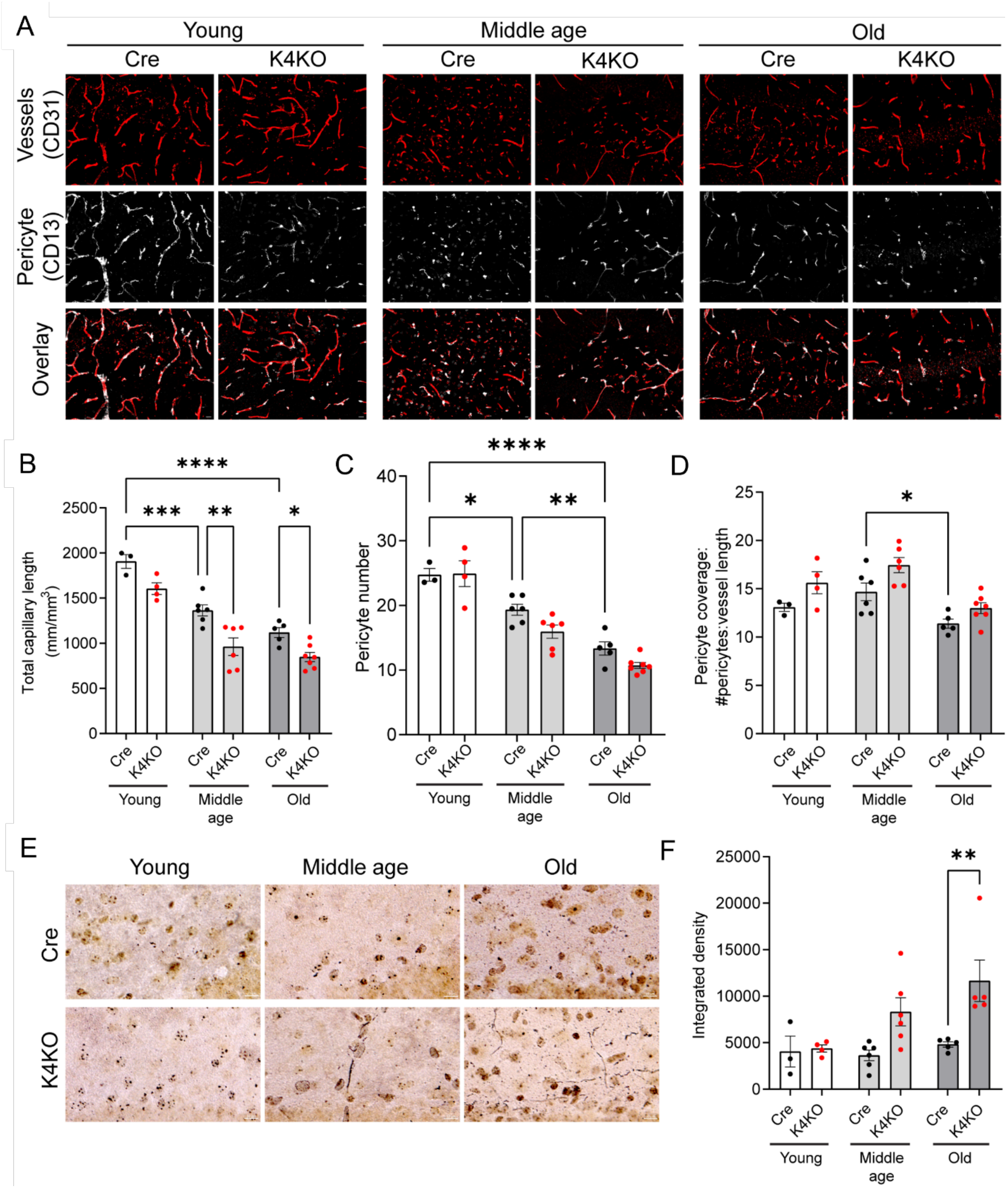

Fig. S5. Loss of endothelial KLF4 causes vessel loss and axonal degeneration in the hippocampus beginning at middle-age. Representative images and quantified data are from mouse hippocampus. **a,b** Reduction in total capillary length is seen earlier in the hippocampus

with age in WT Cre mice (middle-aged WT Cre littermates vs young WT Cre littermates, and old WT Cre littermates vs middle-aged WT Cre littermates), while endothelial loss of KLF4 results in significantly reduced vessel length by middle-age (middle-aged EC-K4KO vs middle-aged WT Cre littermate) that is sustained in old-age (old EC-K4KO vs old WT Cre littermate). **c,** Reduction in pericyte number is analogous to reduction in capillary length in WT Cre littermate mice. **d,** Pericyte coverage of capillaries reduces with age in old WT Cre littermate mice compared to middle-aged WT Cre. **e,f,** Significant increase in axonal degeneration is seen in old EC-K4KO mice compared to WT Cre littermates. Data shown are average (±SEM). Individual data points represent individual animals with black filled circles as WT Cre littermates and red closed circles as EC-K4KO mice. Significance was determined using one-way ANOVA and Bonferroni post hoc analysis. *p<0.05, **p<0.01, ***p<0.001, ****p<0.0001

Supplementary Figure 6

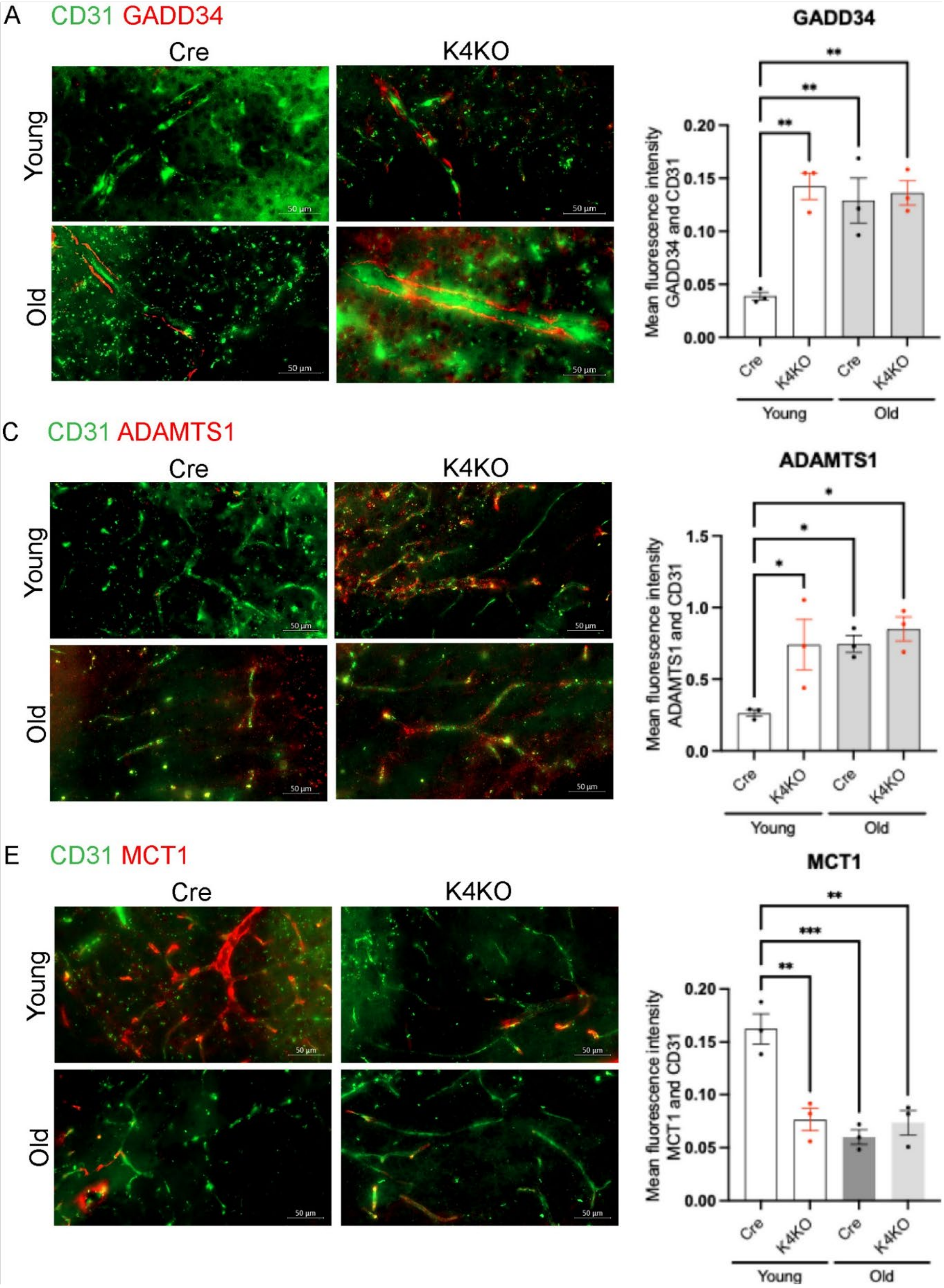

Fig. S6: Differentially expressed genes (DEGs) evaluated at protein level recapitulates aspects of aged EC on young EC-K4KO. Representative images and quantification of the immunofluorescence analysis of the EC marker CD31, with **a,** GADD34 (gene name PPP1R15A), **b,** ADAMTS1, and **c,** MCT1 (gene name SLC16A1), demonstrate that young EC-K4KO mice exhibit a similar protein level pattern to old WT Cre and old EC-K4KO mice, compared to young WT Cre littermates. Data shown are average (±SEM). Individual data points represent individual animals. Significance was tested using one-way ANOVA and Tukey's post hoc analysis for all panels. *p<0.05, **p<0.01, ***p<0.001

SI References

1. K. Kisler, *et al*., *In vivo* imaging an analysis of cerebrovascular hemodynamic responses and tissue oxygenation in the mouse brain. *Nat. Protocols* **13**, 1377-1402 (2018).
2. T.N. Kim, *et al*., Line-scanning particle image velocimetry: an optical approach for quantifying a wide range of blood flow speeds in live animals. *PLOS One* **7**, e38590 (2012).
3. M.M. Janiurek, *et al*., Apolipoprotein M-bound sphingosine-1-phosphate regulates blood-brain barrier paracellular permeability and transcytosis. *Elife* **8**, e49405 (2019).
4. A.C. Ruifrok, D. A. Johnston, Quantification of histochemical staining by color deconvolution. *Anal. Quant. Cytol. Histol.* **23**, 291-299 (2001).
5. M.R. Corces, *et al*., An improved ATAC-seq protocol reduces background and enables interrogation of frozen tissues. *Nat. Methods* **14**, 959-962 (2017).
6. D.R. et al., Kruppel-like factors orchestrate endothelial gene expression through redundant and non-redundant enhancer networks. *J. Am. Heart. Assoc.* **12**, e024303 (2023).
7. H. Li, *et al*., The sequence alignment / map format and SAMtools. *Bioinformatics* **25**, 2078-2079 (2009).
8. Y. Zhang, *et al*., Model-based analysis of ChIP-Seq (MACS). *Genome Biol.* **9**, R137 (2008).
9. F. Ramirez, *et al*., deepTools: a flexible platform for exploring deep-sequencing data. *Nuc. Acids Res.* **42**, W187-W191 (2014).

10. J.T. Robinson, *et al*., Integrative genomics viewer. *Nat. Biotechnol.* **29**, 24-26 (2011).
11. G. Yu, L-G. Wang, Q-Y. He, ChIPseeker: an R/Bioconductor package for ChIP peak annotation, comparison, and visualization. *Bioinformatics* **31**, 2382-2383.
12. W. Wang, *et al*. Exploring epigenomic datasets by ChIPseeker. *Curr. Protoc.* **2**, e585 (2022).
13. G. Yu, *et al*. clusterProfiler: an R package for comparing biological themes among clusters. *OMICS* **16**, 284-287 (2012).
14. G. Yu, Q-Y. He, ReactomePA: an R/Bioconductor package for reactome pathway analysis and visualization. *Mol. Biosyt.* **12**, 477-479 (2016).
15. V. Jalili, *et al*., Using combined evidence from replicates to evaluate ChIP-seq peaks. *Bioinformatics* **31**, 2761-2769 (2015).
